# Rigidified and Hydrophilic DOTA-like Lanthanoid Ligands: Design, Synthesis, and Dynamic Properties

**DOI:** 10.1021/acs.inorgchem.2c03768

**Published:** 2023-02-17

**Authors:** Qing Miao, René Dekkers, Karthick Babu Sai Sankar Gupta, Mark Overhand, Rubin Dasgupta, Marcellus Ubbink

**Affiliations:** † Leiden Institute of Chemistry, Gorlaeus Laboratories, Leiden University, Einsteinweg 55, Leiden 2333 CC, The Netherlands; ‡ College of Chemistry and Chemical Engineering, Key Laboratory of Chemical Additives for China National Light Industry, Shaanxi University of Science and Technology, Xi’an 710021, China; § Department of Medical Biochemistry and Biophysics, Karolinska Institutet, Solnavägen 9, Stockholm 17177, Sweden

## Abstract

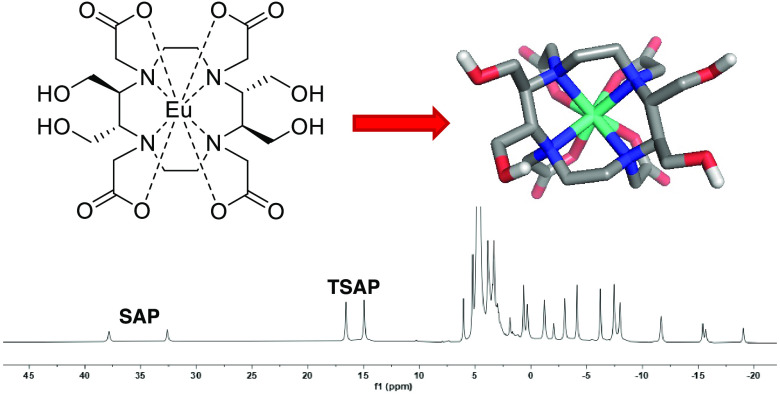

Limiting the dynamics of paramagnetic tags is crucial for the accuracy of the structural information derived from paramagnetic nuclear magnetic resonance (NMR) experiments. A hydrophilic rigid 2,2′,2″,2‴-(1,4,7,10-tetraazacyclododecane-1,4,7,10-tetrayl)tetraacetic acid (DOTA)-like lanthanoid complex was designed and synthesized following a strategy that allows the incorporation of two sets of two adjacent substituents. This resulted in a *C*_2_ symmetric hydrophilic and rigid macrocyclic ring, featuring four chiral hydroxyl-methylene substituents. NMR spectroscopy was used to investigate the conformational dynamics of the novel macrocycle upon complexation with europium and compared to DOTA and its derivatives. The twisted square antiprismatic and square antiprismatic conformers coexist, but the former is favored, which is different from DOTA. Two-dimensional ^1^H exchange spectroscopy shows that ring flipping of the cyclen-ring is suppressed due to the presence of the four chiral equatorial hydroxyl-methylene substituents at proximate positions. The reorientation of the pendant arms causes conformational exchange between two conformers. The reorientation of the coordination arms is slower when the ring flipping is suppressed. This indicates that these complexes are suitable scaffolds to develop rigid probes for paramagnetic NMR of proteins. Due to their hydrophilic nature, it is anticipated that they are less likely to cause protein precipitation than their more hydrophobic counterparts.

## Abstract 2

We report the design and synthesis of a rigidified and hydrophilic DOTA-like lanthanoid ligand, along with its conformational analysis using NMR spectroscopy. A synthetic strategy was described that allows the synthesis of a novel functionalized DOTA derivative.

## Introduction

Due to their physiochemical properties, trivalent lanthanoid ion complexes are widely used in magnetic resonance imaging (MRI) as contrast agents, in nuclear magnetic resonance (NMR) spectroscopy as resonance shift or line-broadening agents, and as luminescent probes.^1−3^ DOTA (2,2′,2″,2‴-(1,4,7,10-tetraazacyclododecane-1,4,7,10-tetrayl)tetraacetic acid) and its derivatives are among the most applied lanthanoid chelators, valued for their high metal binding affinity and the stability of the complexes.^4,5^ The crystal structures of DOTA lanthanoid complexes show that the four nitrogen atoms of the cyclen ring and four oxygen atoms of the pendant arms are involved in metal ion coordination to form a nearly perfect square antiprism.^6−8^ The coordinating nitrogen and oxygen atoms are defined as the N and O planes, respectively. In DOTA lanthanoid complexes, the metal ion is wedged in between these two planes. It was demonstrated that the relative torsion of the two planes yields two distinct coordination geometries of the complexes, referred to as square antiprismatic (SAP) and twisted square antiprismatic (TSAP).^6−8^ The TSAP has a torsion angle of about 25°, while the SAP has a torsion angle of about −39°, thus forming a smaller metal coordination cavity and a more compact structure.^9−11^ NMR studies showed that the ratio of these two conformers of DOTA and its derivatives is dependent on the nature of the lanthanoid ion and the structure of the ligands.^12,13^ These conformers are in exchange on a timescale of 10–100 ms.^9,14,15^ Exchange is caused by two structural changes, a coherent rotation of the pendant arms and a coherent flip of the ethylene groups in the cyclen ring. Consequently, lanthanoid ion DOTA complexes adopt four conformers, two mirror images of SAP and two of TSAP. When the complex is part of a protein tag, these conformers gives rise to multiple resonances for the same nuclei in proteins, thereby hampering application of paramagnetic NMR probes in protein structure and dynamics characterization.^16^ In paramagnetic relaxation dispersion NMR spectroscopy, the exchange between the conformers leads to undesired line-broadening effects that interfere with the analysis of protein dynamics.^17−21^

In addition to conformational exchange, the dynamics of the water in the ninth coordination of the lanthanoid is relevant. The exchange rate of this coordinated water is an important factor for the efficiency of an MRI contrast agent.^1,22−25^ It has been reported that the water exchange rate of the TSAP conformer is about two orders of magnitude faster than that of SAP, making it the preferred conformation for MRI contrast agents.^23−27^ In contrast, for the application as a luminescent probe, the absence of this water molecule is crucial as it can decrease the excitation lifetime of Ln(III).^28^ For the application of the complex in paramagnetic NMR spectroscopy on proteins, which is the focus of this study, the water dynamics is not relevant because it is fast on the applicable NMR timescale, determined by the lanthanoid-induced pseudocontact shifts.^29^

**Chart 1:**
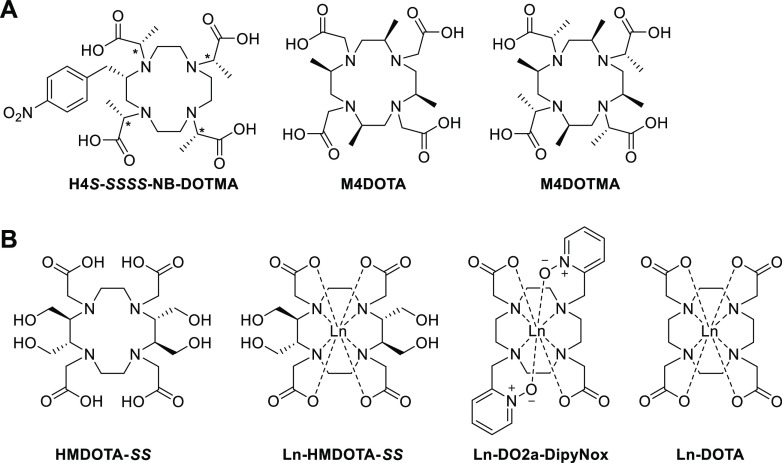
(A) Structure of DOTA-like Rigid Ligands^24,30,31^ and (B) Structures of Lanthanoid Complexes of HMDOTA, DO2A-DipyNox,^32−34^ and DOTA^15,35^ Studied in This Worka

Many efforts have been reported to decrease the number of conformers in DOTA complexes, among which rigidification is the most efficient and frequently employed. The incorporation of bulky groups or chiral carbons on the pendant arms, macrocyclic ring, or both has been previously reported.^24,36−45^ The modification or replacement of the acetate pendant arms has been applied more frequently than the incorporation of specific substituents on the tetraaza ring. The combined modification of pendant arms and ring substituents can result in a highly rigidified DOTA-like ligand. Bulkier group on the ligand however can hinder the lanthanoid ion coordination. By introducing a chiral carbon with a *p*-nitrobenzyl substituent to the tetraaza ring of DOTMA (Chart 1A, H4S-*RRRR*/*SSSS*-NB-DOTMA), Woods et al. reported successful locking the coordination geometry of Eu(III) complexes.^24^ Depending on the chirality of the methyl substituents on the pendant arms, the complexes form either the SAP or TSAP conformer.^24^ In further work, the influence of the chirality of the *p*-nitrobenzyl substitution on the stability and conformational behavior of the complex was studied.^26,46−48^ Various cyclen derivatives have been synthesized that contain substituents on each of the four ethylenediamine moieties, resulting in a complex with *C*_4_ symmetry.^30,40,44^ Among them, tetramethylated cyclen (4MDOTA, Chart 1A) was reported to increase rigidity,^31^ although even with additional chiral methyl modifications on the arms (4MDOTMA, Chart 1A), two isomers are still observed.^49,50^ In recent work, the methyl substituents on the ring were replaced with various other alkyl groups (R = ethyl, benzyl, isobutyl, hydroxyethyl, and butylamine), but no substituents on the pendant arms were included.^40^ It has been reported that the ratio of the two conformers observed for these derivatives depends on the size of the substituents.^40^ The incorporation of alkyl substituents rendered the above-mentioned DOTA derivatives more hydrophobic, which enhances the chance of protein precipitation when used as a probe for paramagnetic NMR.

a The asterisks in the structure of H4S-*SSSS*-NB-DOTMA indicate the asymmetric carbons. Inversion of stereochemistry depicts H4S-*RRRR*-NB-DOTMA.

Here, we report the design and synthesis of a rigidified and hydrophilic DOTA-like lanthanoid ligand, along with its conformational analysis using NMR spectroscopy. We designed a *C*_2_ symmetric chiral cyclen-like ring with two pairs of hydroxyl-methylene substituents, HMDOTA (Chart 1B). Paramagnetic tags that allow protein attachment via two linkers have been reported before.^16,32,33,51−56^ To avoid different conformers upon attachment, the *C*_2_ symmetry of the molecule is a requirement. To investigate the influence of these substituents on the ring flip, the regular pendant arms (tetraacetic acid) were used. Exchange spectroscopy (EXSY) experiments demonstrated that the tetraaza ring of Ln-HMDOTA is rigid. Two exchanging conformers are observed, which are attributed to the reorientation of the pendant arms. The rate of exchange between the conformers is slower for the lanthanoid complex of HMDOTA than for those of the DOTA derivative DO2A-DipyNox (Chart 1B) and DOTA.

## Results and Discussion

### Design and Synthesis

**Scheme 1:**
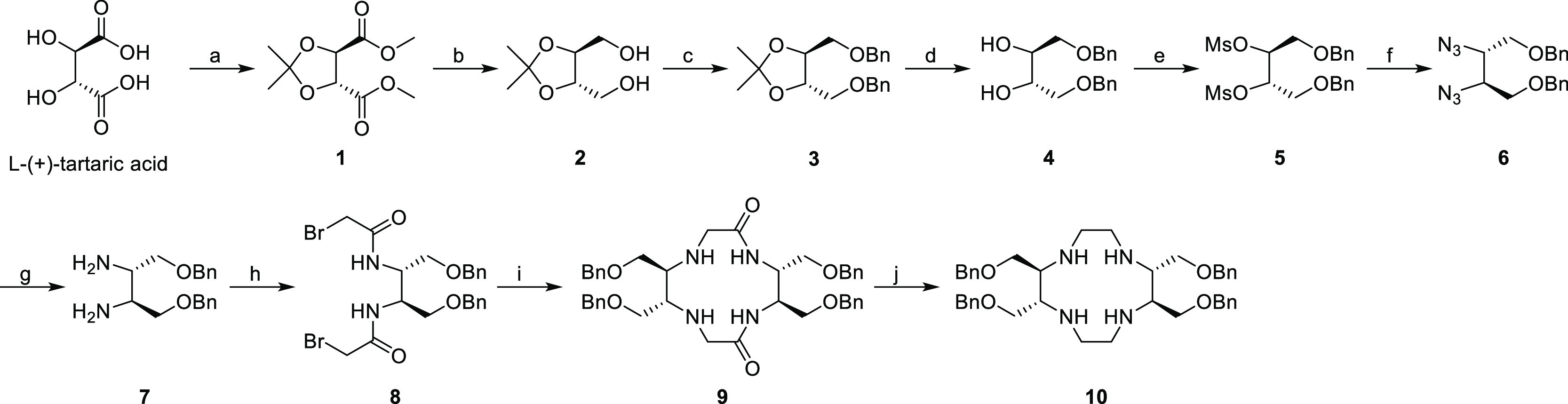
(a) MeOH, DMP, *p*-TsOH, Cyclohexane, 70 °C, 96 h; (b) NaBH_4_, MeOH, rt, 8 h; (c) NaH, BnBr, DMF, rt, 16 h; (d) 1 M HCl, EtOH, 80 °C, 6 h; (e) MsCl, Hünig’s Base, DCM, rt, 3 h; (f) NaN_3_, DMSO, 80 °C, 24 h; (g) Pd/C, H_2_, rt, 24 h; (h) Bromo Acetyl Bromide, K_2_CO_3_, DCM, 0 °C, 10 h; (i) 7, NaHCO_3_, ACN, 80 °C, 10 h; and (j) Red-Al, Toluene, 80 °C, 24 h

In our design, it was anticipated that the introduction of two substituents on adjacent carbons of the cyclen-ring system with the appropriate stereochemistry relative to each other would position them both equatorial upon complexation with a metal. To ensure *C*_2_ symmetry, a second set of substituents on the opposite side of the cyclen-ring system was incorporated. The hydroxymethyl group was selected to increase the hydrophilicity of the complexes. A previously reported cyclization method was applied with improvements.^44,57^l-(+)-Tartaric acid was modified via standard procedures to obtain the derivatives **7** and **8**, amenable to cyclization (Scheme 1). To decrease side product formation due to intermolecular reactions of this key step, the base, the concentration, and the temperature of the cyclization reaction were optimized.

**Scheme 2:**
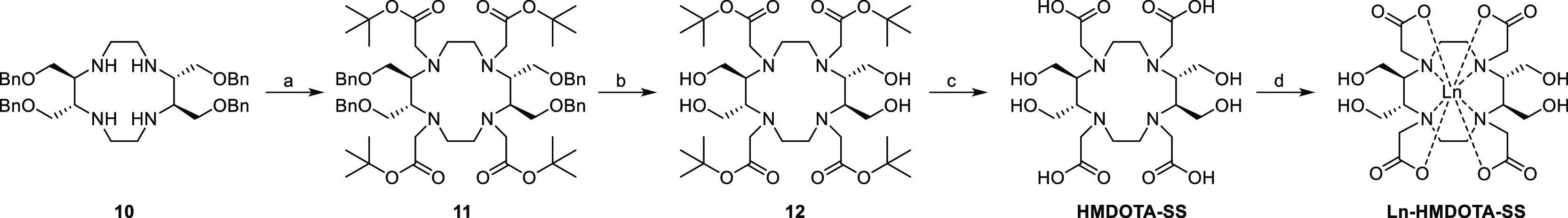
(a) *t*-Butyl Bromoacetate, K_2_CO_3_, ACN, rt, 16 h; (b) Pd/C, H_2_, rt, 72 h; (c) TFA/DCM (4:1), 0 °C, 16 h; and (d) LnCl_3_, D_2_O, pH = 8, rt

The chance of intermolecular reactions was reduced by performing the cyclization at a low concentration. It was found that concentrations below 0.03 mol/L did not result in a further increase in the yield of the desired cyclized product. According to the liquid chromatography-mass spectrometry (LC–MS) analysis, the main side product was the over-alkylated product formed between cyclized compound **9** and compound **8** (Scheme S1). The effects of temperature and acid scavenger were evaluated as shown in Table S1. When the reaction was performed at room temperature with NaHCO_3_ as the base the lowest yield for both products was obtained, while at 80 °C most cyclized compound was produced. In comparison with the other acid scavengers, NaHCO_3_ is a weak base, thus it may be that both alkylation reactions are slow at room temperature, whereas at high temperature the intramolecular *N*-alkylation is preferred over the intermolecular alkylation of the secondary amine group. Therefore, the cyclization was performed at a concentration of 0.03 mol/L, at a temperature of 80 °C, and using NaHCO_3_ as the base. The acquired cyclic diamide **9** was treated with the reagent Red-Al to reduce the two amide groups to amines. Compound **10** was peralkylated with *tert*-butyl bromoacetate in the presence of K_2_CO_3_ at room temperature (Scheme 2). Removal of the protective groups provided HMDOTA, which was used to prepare the metal complexes.

Chloride salts of lanthanoids were used for the preparation of all metal complexes. For HMDOTA, the formation of the complexes was performed in D_2_O at pH 8 and room temperature for 12 h. The Eu(III) loaded HMDOTA has similar NMR spectra at different temperatures and pD values (pD = pH + 0.4) 2.4 and 12.4, as shown in Figure S1. For comparison, lanthanoid complexes of DOTA (commercially purchased) and a previously reported DOTA derivate (DO2A-DipyNox^34^,^45^) were prepared as well. A detailed description of the synthesis is given in the Materials and Methods.

### Conformational Dynamics

**Figure 1:**
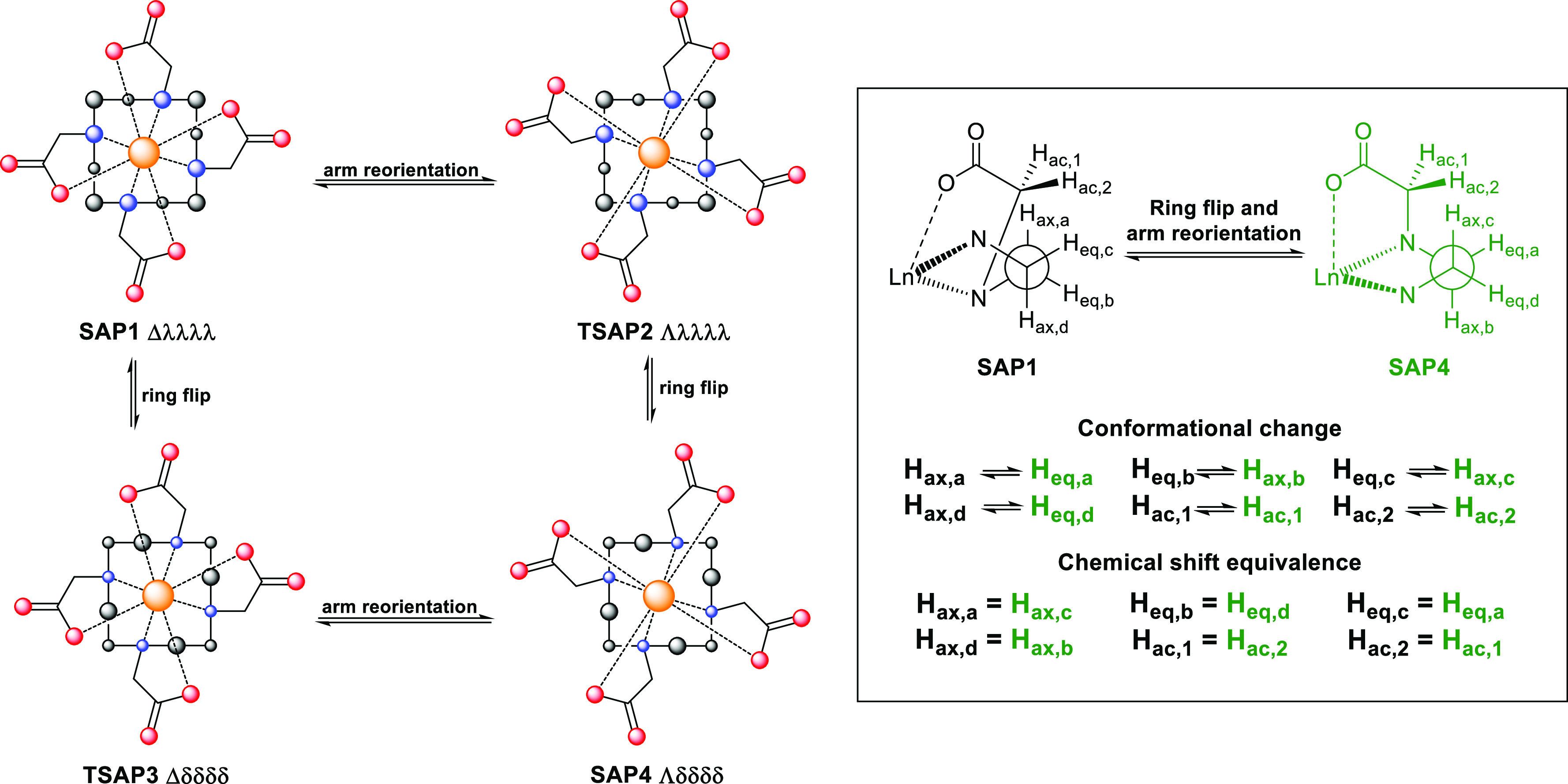
Schematic representation of DOTA lanthanoid complex conformation exchange (left) and the effect of the ring flip and arm reorientation exchange processes on different protons (right). The cyclen ring is shown as a square in solid lines; nitrogen, oxygen, and carbon atoms of DOTA are shown as blue, red, and black spheres, respectively; and the metal ion is shown as a brown sphere.

**Figure 2:**
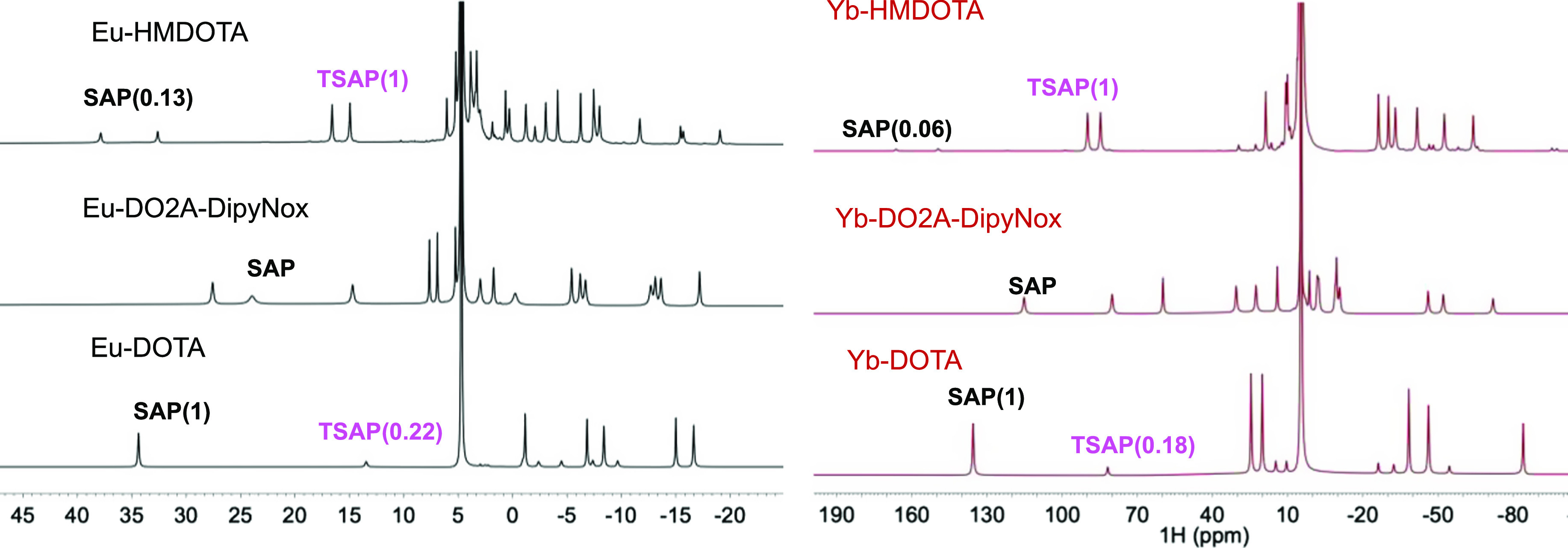
^1^H NMR spectra of Eu(III) and Yb(III) loaded HMDOTA, DO2A-DipyNox, and DOTA recorded at 20 °C and 14.1 T (600.130 MHz). The relative integrals of the peaks for the SAP and TSAP isomers are given in brackets.

The change in the torsion of the N- and O-planes represents a conformational exchange of the DOTA complex, due to the reorientations of the pendant arms and the flip of the cyclen ring. The coordination of the carboxylic pendant arms can rotate either clockwise (Λ) or anticlockwise (Δ) and the tetraaza macrocyclic ring can adopt λλλλ or δδδδ conformations. This results in four conformations present as two mirror-image pairs: Δ(λλλλ) and Λ(δδδδ) in one, Λ(λλλλ) and Δ(δδδδ) in another (Figure 1). The Δ(λλλλ) and Λ(δδδδ) are the SAP conformers, whereas the Λ(λλλλ) and Δ(δδδδ) are the TSAP conformers.^10,11^ These two geometries differ in their magnetic properties if the bound metal ion is paramagnetic, enabling the conformers to be distinguished by NMR spectroscopy. The 1D ^1^H spectra of Eu(III)-DOTA and Yb(III)-DOTA (Figure 2) are in agreement with previous reports.^11,58^ Two sets of six resonances each were observed for Eu(III)-DOTA and Yb(III)-DOTA. These two sets correspond to the resonances of the SAP and TSAP conformers.^24,25,40,58^ The mirror image conformers have identical NMR spectra. The relative intensities of the SAP and TSAP resonances in the 1D ^1^H spectra indicate that the SAP conformer is dominant in the DOTA complexes.

**Figure 3:**
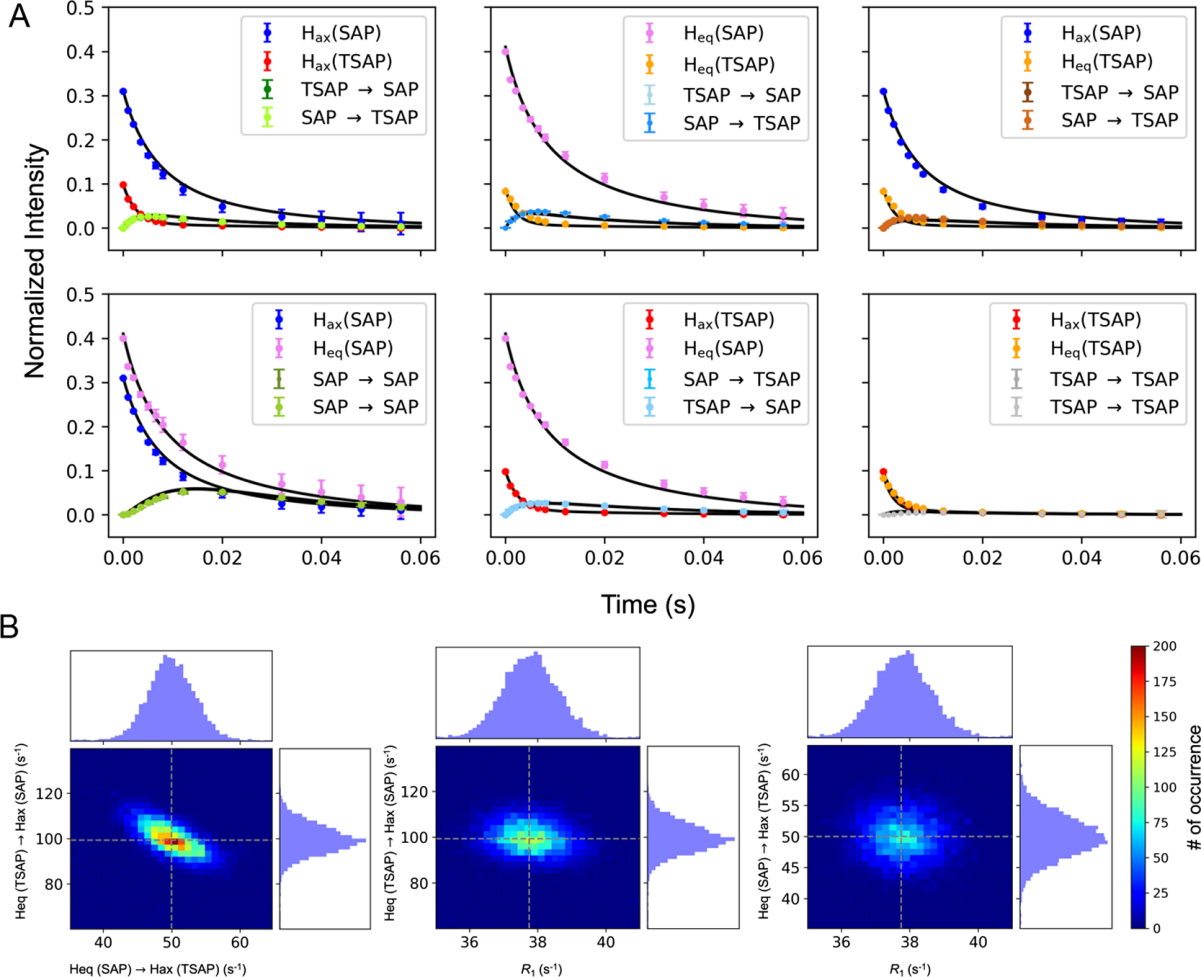
(A) Mixing time dependence of signal intensities of diagonal and cross peaks from ^1^H-^1^H EXSY of the resonances H_ax_ (SAP), H_ax_ (TSAP), H_eq_ (SAP), and H_eq_ (TSAP). Each conformer is color coded and is shown in the legends on the graph. The data are shown as filled circles and the fit to eq 7 is shown as the solid black line. The errors in the data are estimated from the noise level. The reduced χ^2^ of the fit is 0.97. (B) Monte-Carlo distribution and correlations between each fit parameter and the constrained longitudinal relaxation rate *R*_1_ (38 ± 1 s^–1^). The histogram distribution is shown for each fitted parameter. The dotted line is the best-fit value of the fit parameters. The color bar shows the density of the Monte-Carlo samples.

To study the exchange process between the conformers, EXSY experiments^59,60^ were recorded with mixing times varied from 0 to 56 ms. It has been reported that exchange occurs in DOTA with the rate of about 100 s^–1^ between the four conformers (Figures 1 and S2).^9,14,15^ Due to the large shift induced by the paramagnetic metal ion, the chemical shift difference (expressed in rad/s) for a nucleus in the two conformers is much larger than 100 s^–1^, so the exchange is slow on the NMR timescale, yielding separate signals for the different conformers. Exchange on the millisecond timescale between the four conformers is evident from the presence of cross-peaks in the EXSY spectrum. The diagonal and cross-peak intensities in the EXSY spectra are dependent on the mixing times and can be fitted to estimate the exchange rates between conformers. Note that the EXSY experiment can also reveal an exchange between the mirror-image conformers. Though the 1D spectra of these conformers are identical, the nuclei that cause the given resonances interchange when transiting between the mirror images, for example, an axial proton becomes the equivalent equatorial proton and vice versa (see the right panel in Figure 1). The ^1^H-^1^H EXSY spectrum of Eu(III)-DOTA shows the presence of all four exchanging conformers (Figure S2). The resonances used for each of the conformers to estimate the exchange rate are from H_ax,a_ (SAP), H_ax,a_ (TSAP), H_eq,a_ (SAP), and H_eq,a_ (TSAP), as shown in Figure S2 (the subscript a is removed for the conformational dynamics analysis). The exchange nature of the cross-peaks (rather than NOE) was confirmed by the temperature dependence of the cross-peak intensities (Figure S2D). The exchange model is restrained by the fact that the four states represent two pairs of mirrored molecules, resulting in a single pair of forward and backward rate constants for arm rotation and a single pair for ring flip. Furthermore, the equilibrium constants of both processes are the same and can be derived from the 1D NMR spectra (Figure 2, Table S2). These restraints result in only two degrees of freedom for the fitting of the rate constants. Additionally, the longitudinal relaxation rate (*R*_1_) was estimated to be 38 ± 1 s^–1^ for all four signals using an inversion recovery experiment (Figure S2E) and was constrained between 36 and 39 s^–1^ for the Bloch–McConnell (BM) matrix fitting. The exchange rates and the errors estimated from the Monte-Carlo simulation (Figure 3) are summarized in Table 1. Previously, the exchange rates in the Eu(III)-DOTA were measured at 278 K (Table 1). Here, the experiment was done at 293 K, explaining the higher exchange rates. In one report, the exchange rate for the arm rotation was about half that of the ring flip,^58^ whereas in the other it is about double,^61^ suggesting that in one report the processes were annotated wrongly. Our data show arm rotation is faster than ring flipping, in line with the latter report.

**Table 1:** Rate Constants for Ring Flip and Arm Rotation in Eu(III)-DOTA-like Compounds^a^

compound	motion	isomer	rate constants (s^–1^)	exchange rate^b^ (293 K) (s^–1^)	exchange rate (278 K) (s^–1^)
DOTA	arm rotation	SAP ⇀ TSAP	48 ± 4	239	31,^e^ 78^f^
		TSAP ⇀ SAP	191 ± 13		
	ring flip	SAP ⇀ TSAP	24 ± 2	119	63^e^, 35^f^
		TSAP ⇀ SAP	95 ± 6		
	arm rotation + ring flip	SAP ⇀ SAP	16^c^	32	7,^c^^,^^e^ 23^f^
		TSAP ⇀TSAP	63^c^	127	35^c^^,^^e^
DO2A-DipyNox	arm rotation + ring flip	SAP ⇌ SAP	88	177 ± 2	
HMDOTA	arm rotation	SAP ⇀ TSAP	37^d^	44 ± 6	
		TSAP ⇀ SAP	7^d^		

a The error is the 1σ value from the Monte-Carlo simulation and is given for the experimentally determined rate constants or exchange rates.

b *k*_ex_ = *k*_forward_ + *k*_backward_.

c Calculated with eqs 4 and 5.

d Based on *p*_TSAP_ = 0.84, *p*_SAP_ = 0.16.

e Calculated with rate constants from ref (58).

f Reported in ref (61).

**Figure 4:**
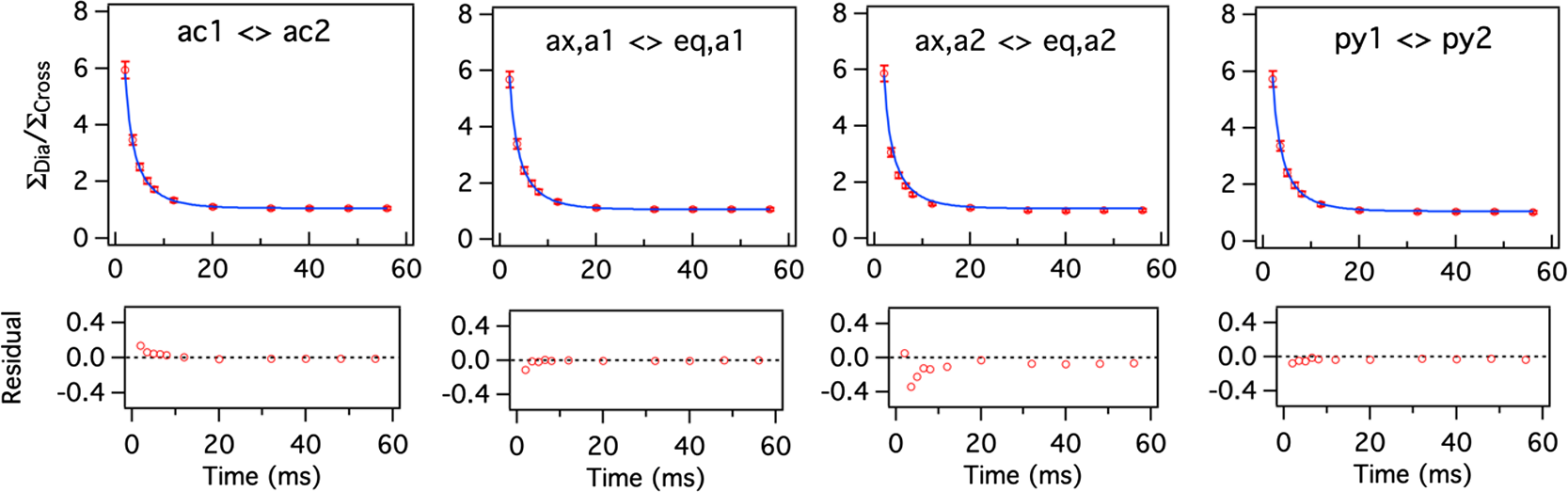
Global fit to eq 12 for the exchanging pairs of ac1 ⇌ ac2, ax,a1 ⇌ eq,a1, ax,a2 ⇌ eq,a2, and py1 ⇌ py2 in Eu(III)-DO2A-DipyNox. The residuals of the fit are shown below the curves. The data points are represented as red circles with the error bars estimated from the noise level. The fit is shown as the blue solid line.

It has been reported that the introduction of pyridine-*N*-oxide arms (DO2A-DipyNox) reduces the number of observable conformers,^33,34^ which was confirmed by the 1D ^1^H spectrum of this compound (Figures 2 and S3). Six pairs of resonances (excluding the peaks of the pyridine-*N*-oxide ring) are observed corresponding to two SAP mirror images that are in chemical exchange. This suggests that in Eu(III)-DO2A-DipyNox reorientation of the pendant arms and flipping of the cyclen ring occurs in a concerted fashion with the intermediate TSAP form being very lowly populated, similar to what was reported for CLaNP5 (Caged Lanthanoids NMR probe # 5).^19^ The longitudinal relaxation rate (*R*_1_) for the protons marked as ac1, ac2, ax,a1, ax,a2, eq,a1, eq,a2, py1, and py2, was determined to be 34 ± 1 s^–1^ (Figure S4). Surprisingly, the relaxation rate is the same for the different hydrogens. Since the *R*_1_ is the same for all the protons and the resonance pattern suggests a two-state exchange process, eq 12 can be used to estimate the exchange rate from the ratio of peak integrals of diagonal and cross-peaks with respect to mixing time in the EXSY experiment.^60^ The exchange rate after globally fitting the exchange pairs of ac1 ↔ ac2, ax,a1 ↔ eq,a1, ax,a2 ↔ eq,a2, and py1 ↔ py2 was estimated to be 177 ± 2 s^–1^ using a least-square fitting algorithm with population fixed to 50% since both the SAP conformers are mirror images (Figure 4, Table 1).

**Figure 5:**
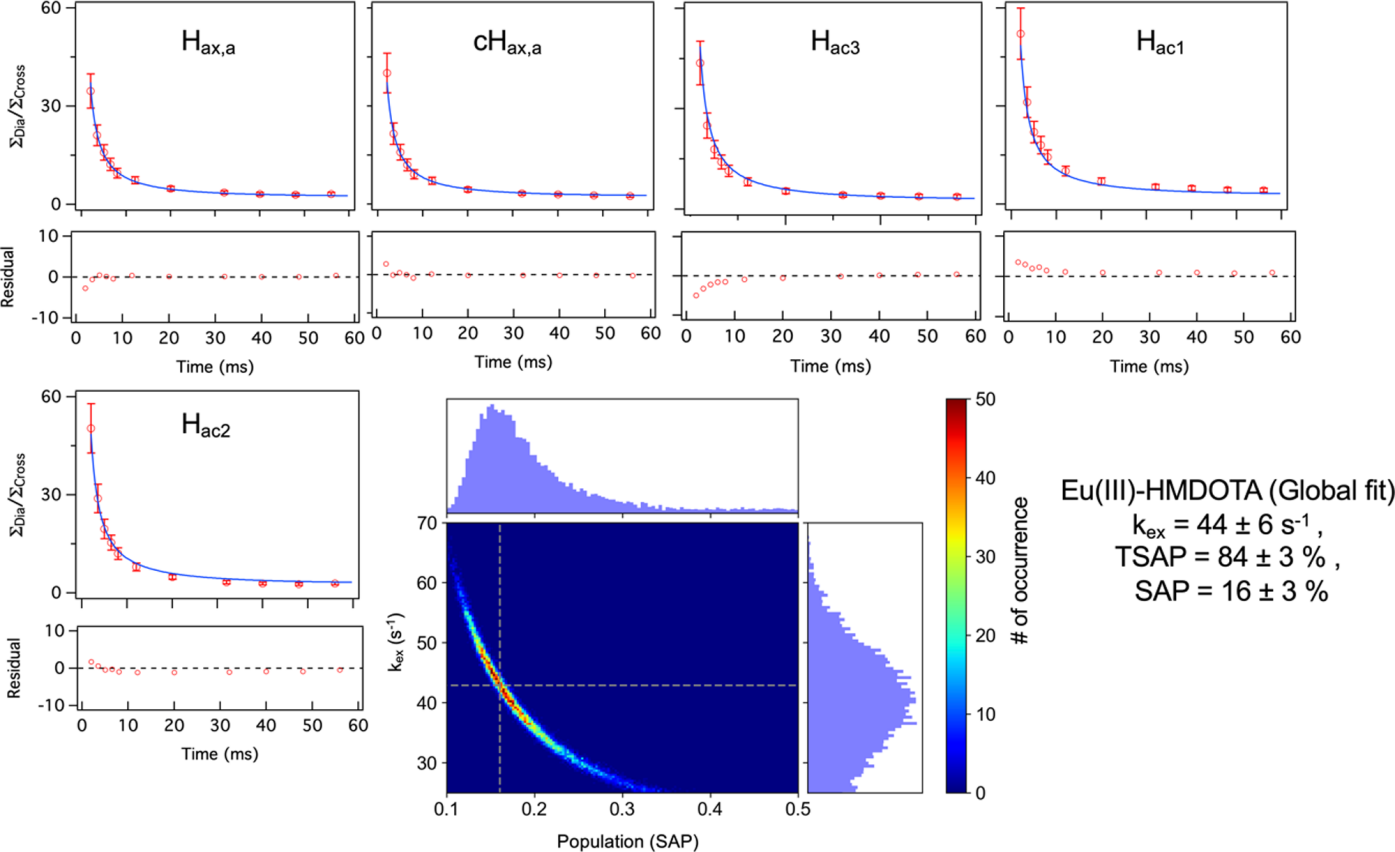
Global fit to eq 12 to estimate the exchange rate for the exchanging pairs between TSAP and SAP conformers for protons H_ax,a_, cH_ax,a_, H_ac1_, H_ac2_, and H_ac3_ in Eu(III)-HMDOTA. The residuals of the fit are shown below the curves. The data points are represented as red circles with the error bars estimated from the noise level. The fit is shown as a blue solid line. Monte-Carlo simulation of the global fit is also shown. The color bar shows the samples of the MC simulation. The dotted gray lines represent the best-fit value. The histograms show the distribution of the values for exchange rate and population. Best fit parameters and the errors from the MC simulation are reported.

For Eu(III)-HMDOTA, it is found that TSAP is the major conformer, contrary to Eu(III)-DOTA, for which the SAP conformer is dominant (Figure 2). Similar results were found for Yb(III) loaded HMDOTA and DOTA. The ratio of the relative integrals between SAP and TSAP shows that the SAP conformer in Yb(III) loaded HMDOTA is ∼50% less populated than the Eu(III) loaded complex (Figure 2 and Table S2). This difference in the ratio indicates that the population of the minor conformer may depend on the ionic radius, as well as on the compound, as observed before.^62^ The EXSY spectrum of Eu(III)-HMDOTA shows the presence of one set of exchange cross-peaks corresponding to chemical exchange between SAP and TSAP conformers due to arm rotation (Figure S5). The exchange process was characterized analogously to that of DO2A-DipyNox. It was observed that the exchanging pairs have the same *R*_1_ relaxation rates, however, two *R*_1_ rates are observed, dependent on the proton position. The hydrogens H_ax,a_ and cH_ax,a_ in both TSAP and SAP conformation yield *R*_1_ = 56.5 ± 0.5 s^–1^, while for H_ac,1_, H_ac,2_, and H_ac,3_, *R*_1_ = 28 ± 1 s^–1^ was obtained (Figure S6). The exchange rate was estimated using eq 12(60) yielding 44 ± 6 s^–1^ with populations of 16 ± 3 and 84 ± 3% for SAP and TSAP conformers, respectively (Table 1). The errors were estimated from the Monte-Carlo simulation (Figure 5). These observations show that the hydroxymethyl substituents reduce the exchange rate for arm rotation significantly compared to that in DOTA.

## Conclusions

**Figure 6:**
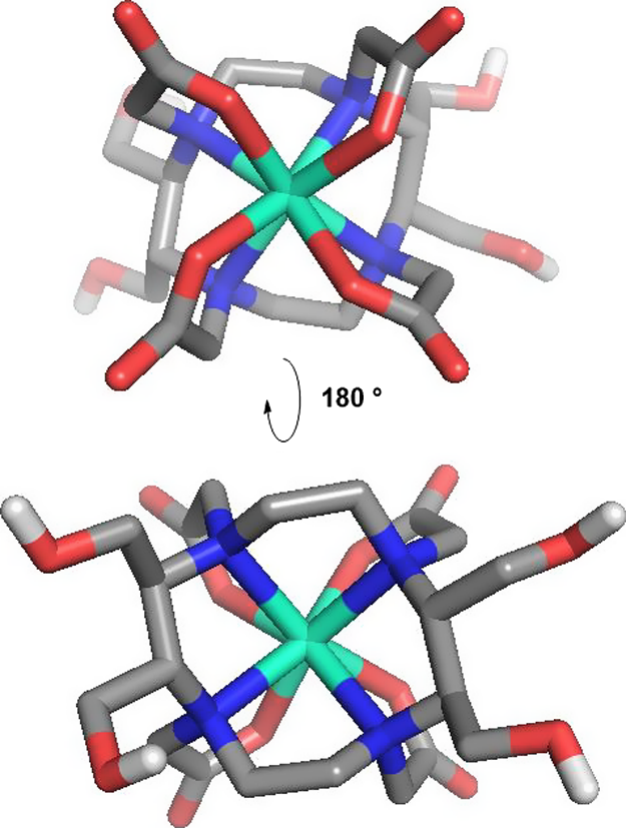
Model of the coordination of Ln(III)-HMDOTA as deduced from NMR studies and molecular conformational distribution calculations. Carbons are shown in gray, nitrogen in blue, oxygen in red, hydrogens in white, and Ln(III) in mint, respectively.

A synthetic strategy was described that allows the synthesis of a novel functionalized DOTA derivative with substituents on the tetraaza ring at adjacent positions. In our design, we anticipated that the introduction of two substituents on adjacent carbons of the cyclen-ring system with the appropriate stereochemistry relative to each other would, upon chelation, position them both equatorial in the 12-membered heterocyclic ring. To enhance this effect and ensure *C*_2_ symmetry, a second set of substituents on the opposite side of the ring system was incorporated. Because of the hydroxymethyl substituents, this DOTA derivative is expected to be more hydrophilic. Figure 6 shows a model of Ln(III)-HMDOTA, in which it can be seen that the cyclen ring is bulkier due to the substituents but also that the hydroxy groups add polarity to the more hydrophobic side of the complex, below the N-plane. Both SAP and TSAP conformers exist, with a higher abundance of TSAP after ligation to medium- or small-size lanthanoids. EXSY experiments indicate that conformational exchange is limited to the rotation of the flexible acetate arms. The rate of exchange is reduced by a factor of 5 compared to arm rotation in DOTA. For application in paramagnetic relaxation NMR, it is necessary to rigidify the complex further, for which modification of the pendant arms is a logical next step. For example, the introduction of chiral centers or replacing the acetic acid arms with pyridine-oxide arms will be interesting to analyze. The results with DO2A-DipyNox indicate that the latter group promotes the stability of one conformer over the other. Such a compound could find application in structural and dynamics studies of proteins.

## Materials and Methods

All the chemicals were purchased from Sigma-Aldrich, Merk, Fisher Scientific, or VWR and used as received. DOTA was purchased from MacrocyclicsTM. DO2A-DipyNox is adopted from our previous studies,^32,33^ and was synthesized by Dr. Liu (present address: Fu Jen Catholic University, New Taipei City, Taiwan). Solvents were purchased from Honeywell, BIOSOLVE or Aldrich and stored over 3 Å molecular sieves before use. Traces of water from reagents were removed by co-evaporation with toluene in reactions that required anhydrous conditions. Flash chromatography was performed on Screening Devices silica gel 60 (40–63 μm) and C18-reversed phase silica gel (fully endcapped, 40–63 μm). LC–MS analysis was performed on a Surveyor HPLC system (Thermo Finnigan) equipped with a C18 column (Gemini, 4.6 mm × 50 mm, 5 μm particle size, Phenomenex), coupled to an LCQ Advantage Max (Thermo Finnigan) ion-trap spectrometer (ESI+). Thin-layer chromatography (TLC) analysis was performed on a silica gel (F 1500 LS 254 Schleicher and Schuell, Dassel, Germany), which was visualized by UV and/or ninhydrin, KMnO_4_. Reactions were monitored by LC–MS analysis and TLC analysis. Waters preparative HPLC system, equipped with a Waters C18-Xbridge 5 μm OBD (30 × 150 mm^2^) column, was used for purification, and the applied buffers were H_2_O (2% TFA) and ACN. High-resolution mass spectrometry analysis was performed with an LTQ Orbitrap mass spectrometer (Thermo Finnigan), equipped with an electron spray ion source in a positive mode (source voltage 3.5 kV, sheath gas flow 10 mL/min, and capillary temperature 250 °C) with resolution *R* = 60,000 at *m*/*z* 400 (mass range *m*/*z* = 150–2000) and dioctyl phthalate (*m*/*z* = 391.284) as a “lock mass.” NMR spectra were recorded on a Bruker AV-400 (400.130/100.613 MHz), AV-500 (500.130/125.758 MHz), or AV-HDIII-600 (600.130/150.903 MHz) spectrometer. Chemical shifts (δ) are reported in ppm relative to the residual signal of the deuterated solvent.

### Metal Complex Preparation

Compound HMDOTA (3 mg, 5.7 μmol) was dissolved in D_2_O at room temperature and the pD value was adjusted to around 8 by dropwise addition of 1 M NaOD. An equal amount of LnCl_3_·*n*H_2_O was added to this solution, and the pD value was adjusted to 8 again adding 1 M NaOD dropwise. After the reaction mixture was continuously stirred at room temperature for 12 h, the thus obtained lanthanide complex solutions were directly used for NMR measurements. The same procedure was used for the preparation of the Ln-DOTA and Ln-DO2A-DipyNox complexes.

### NMR Spectroscopy

All the EXSY and 1D ^1^H NMR spectra were recorded at 600 MHz NMR spectrometer equipped with cryoprobe TCI 600 H&F/C/N-D-05 Z-gradient at 293 K unless otherwise specified. For EXSY experiments, the mixing time ranged from 0 to 56 ms. The recovery delay in the inversion recovery experiment ranged from 0.1 to 300 ms with an interscan delay set to 200 ms. Data were processed and analyzed using a Bruker Topspin 3.6.3, Igor Pro 6.3.6, and an inhouse Python 3.9 script.

### BM Numerical Fitting of the EXSY

The BM numerical method was used to fit the integral volume of the diagonal and the cross-peaks with respect to the mixing time for Eu(III)-DOTA. The four-state model is shown in Figure 1. For simplicity, the following definition is used for the derivation of the rate equations: A = H_ax_ (SAP), B = H_ax_ (TSAP), C = H_eq_ (SAP), and D = H_eq_ (TSAP) (Figure S2A, note that the subscript “a” is removed). Here, A ⇌ B and D ⇌ C are in exchange due to arm rotation, while B ⇌ C and A ⇌ D are in exchange due to ring flip, as shown in Figure 1. Additionally, two SAP or TSAP conformers are mirror images, hence *k*_AB_ = *k*_CD_ and *k*_BC_ = *k*_DA_, and analogously for the back-rates. The exchange rates for ring flip and arm rotation can be different. The equilibrium constant *K*_eq_ (TSAP/SAP) between the SAP and TSAP conformers can be used to reduce the parameter space for fitting via the following relations
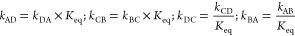
1

For the rate constants that describe the conversion from a conformer into its mirror image (*k*_AC_, *k*_CA_, *k*_DB_, and *k*_BD_), there are two pathways for each.
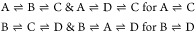
2

*k*_BD_ and *k*_AC_ can be represented as the linear combination of the probability from each of the pathway in eq 2
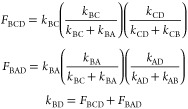
3

Similarly,
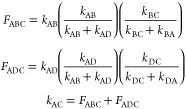
4

The rate constants *k*_CA_ and *k*_DB_ can be obtained in the similar way. These relationships between the rate constants result in only two free-floating fit parameters *k*_CD_ and *k*_DA_ and the *R*_1_ relaxation rate that is restrained between the range estimated from the inversion recovery experiment (Figure S2E). The above-mentioned rate constants were used to build a BM matrix for the four-state exchange as shown below.
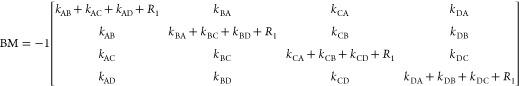
5

The initial intensities of the four state are given as a vector. This is the population of each state at 0 ms mixing time of the EXSY experiment.
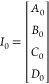
6

The matrix is propagated for the given mixing time (τ_mix_), and differential eq 7 is solved to get the intensity of the diagonal and the cross-peaks after each mixing time. These intensities are fitted to the experimental value using least square fitting (“leastsq”) as implemented in the minimizer package of python 3.9. The Markov Chain Monte Carlo (MCMC) algorithm as implemented in the ‘emcee’ package of python 3.9 was used for the error estimation of the fit parameters. In total, 64 walkers with 5000 steps were run for the MCMC. In total, 500 samples were discarded from the start of the sampling regime. Plotting of the heat map was done using a thin sample of 50.
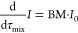
7

### Analytical Solution for a Two-Site Exchange

For a system showing a two-site exchange and with the spin–lattice relaxation rates (*R*_1_) that are the same for both states, the integrals of the cross-peaks and diagonal peaks are given by.^60^
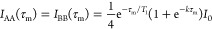
8
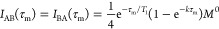
9
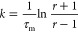
10
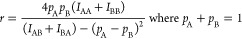
11
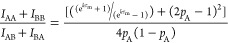
12where *I*_AA_ and *I*_BB_ are the integrals of the diagonal peaks; *I*_AB_ and *I*_BA_ are the integrals of the cross-peaks, τ_m_ is the mixing time, *k* is the exchange rate constant, and *p*_A_ and *p*_B_ are the mole fractions of the two states, respectively. Equation 12 was used to fit the ratio of diagonal and cross-peak integrals for Eu(III)-HMDOTA and Eu(III)-DO2A-DipyNox complexes.

### Molecule Conformational Modulation

A 3D structure was obtained by conformational distribution calculation with the molecular mechanic of Merck molecular force field.^63^

### Synthesis

#### Dimethyl (4*R*,5*R*)-2,2-Dimethyl-1,3-dioxolane-4,5-dicarboxylate (**1**)

52.33 g of l-(+)-tartaric acid (356 mmol) was dissolved in a mixture of 50 mL of methanol (39.6 g, 1.24 mole) and 25 mL of cyclohexane. 2,2-Dimethoxypropane (DMP, 500 mL, 425 g, 4.08 mole) and *p*-toluenesulfonic acid monohydrate (*p*-TsOH·H_2_O, 0.846 g, 4.45 mmole) were added after which the reaction mixture was heated to 70 °C. The reaction progress was monitored by TLC. After 96 h, the mixture was cooled to room temperature and quenched adding triethylamine dropwise until the pH of the mixture was approximately 8. The mixture was concentrated under reduced pressure using a rotary evaporator and purified using silica gel column chromatography (10:90 → 30:70 EtOAc:*n*-pent, *R*_f_ = 0.65). 54.96 g of compound **1** (254 mmol, 72% yield) was obtained as yellow-colored oil. The spectroscopic data of **1** are in agreement with those reported in the literature.^64^

#### ((4*S*,5*S*)-2,2-Dimethyl-1,3-dioxolane-4,5-diyl)dimethanol (**2**)

A solution of compound **1** (31.54 g, 145 mmol) in methanol (315 mL) was cooled to 0 °C after which NaBH_4_ (16.81 g, 444 mmol) was added in small portions. The reaction mixture was stirred and cooled at 0 °C for another hour after which it was allowed to warm to room temperature and stirring was continued for 8 h. The reaction was cooled in an ice bath and quenched by adding 60 mL of EtOAc. The reaction mixture was concentrated under reduced pressure using a rotary evaporator. Synthesis was continued without further purification of **2**. The crude spectroscopic data were in agreement with those reported in the literature.^65^

#### (4*S*,5*S*)-4,5-Bis((benzyloxy)methyl)-2,2-dimethyl-1,3-dioxolane (**3**)

Crude compound **2** (24.38 g, 150 mmol) was dissolved in 500 mL of DMF and cooled to 0 °C in an ice bath. A 60% oil dispersion of NaH (18.69 g) was added in small portions after which the mixture was stirred for 30 min. 45 mL of benzyl bromide (BnBr, 64.8 g, 379 mmol) was added dropwise. The reaction mixture was slowly allowed to warm to room temperature and stirred for 16 h. Quenching the reaction was done by adding 200 mL of demi water. The mixture was partly concentrated under reduced pressure using a rotary evaporator. The resulting crude reaction mixture was diluted with EtOAc and extracted with water (5 times, 1600 mL each extraction) and brine. The combined organic layers were dried using Mg_2_SO_4_, filtered, and concentrated. The obtained crude product was purified using silica gel column chromatography to obtain compound **3** as a colorless oil with quantitative yield (*R*_f_ = 0.8 in 1:99 EtOAc/*n*-pent). The spectroscopic data of **3** are in agreement with those reported in the literature.^66^

#### (2*S*,3*S*)-1,4-Bis(benzyloxy)butane-2,3-diol (**4**)

A 1 M HCl solution in ethanol was prepared (700 mL) to which compound **3** was added (49.9 g, 146 mmol). The mixture was heated to 80 °C while stirred. After 6 h, the mixture was neutralized by adding saturated aqueous NaHCO_3_ (45 mL) and partly concentrated under reduced pressure. Purification was done using silica gel column chromatography (25% EtOAc/*n*-pent, *R*_f_ = 0.09). Compound **4** was obtained as a white solid with a yield of 84.3% (37.19 g, 123.1 mmol). Spectroscopic data of **4** are in agreement with those reported in the literature.^67^

#### (2*R*,3*R*)-1,4-Bis(benzyloxy)butane-2,3-diyl Dimethanesulfonate (**5**)

12.0 g of compound **4** (40 mmol) was dissolved in DCM (500 mL) and cooled to 0 °C. Hünig’s base (21 mL, 121 mmol) was added dropwise followed by the addition of methanesulfonyl chloride (12.25 mL, 158 mmol). The reaction mixture was allowed to warm to room temperature and stirred. After 3 h, the mixture was cooled to 0 °C and quenched by slowly adding H_2_O (225 mL). The reaction mixture was diluted with DCM (1000 mL), washed twice with demi-water (1500 mL), and once with brine (1500 mL). The organic layer was dried with anhydrous Na_2_SO_4_, filtrated, and concentrated under reduced pressure. Flash silica gel column chromatography with a 20% acetone in *n*-pentane eluent (20:80 Ace:*n*-Pent, *R*_f_ = 0.44) was applied to yield 91% of compound **5** (16.8 g, 36.8 mmol) as a white solid. The spectroscopic data of **5** are in agreement with those reported in the literature.^68^

#### ((((2*S*,3*S*)-2,3-Diazidobutane-1,4-diyl)bis(oxy))bis(methylene))dibenzene (**6**)

Compound **5** (16 g, 35 mmol) was dissolved in DMSO (250 mL). Sodium azide (16 g, 246 mmol) was added in portions after which the reaction mixture was heated for 24 h at 80 °C. The reaction mixture was allowed to cool to room temperature and diluted with DCM (1600 mL), washed with demi-water (two times 1600 mL) and brine (1600 mL). The organic layer was dried with anhydrous Na_2_SO_4_, filtrated, and concentrated under reduced pressure. Further purification was acquired with flash silica gel column chromatography (0:100 → 10:90 EtOAc/*n*-pent, R_f_ = 0.53 for 5:95). Compound **6** was obtained as a yellow-colored liquid with 95% yield (11.71 g, 33.25 mmol). The spectroscopic data of **6** are in agreement with those reported in the literature.^68^

#### (2*S*,3*S*)-1,4-Bis(benzyloxy)butane-2,3-diamine (**7**)

Compound **6** (3 g, 8.60 mmol) was dissolved in methanol (90 mL) and treated with a catalytic amount (10% palladium on carbon) (610 mg). The mixture was stirred for 24 h under a hydrogen atmosphere. The reaction mixture was filtered through Celite and concentrated under reduced pressure. A slightly yellow-colored oil was obtained as compound **7** that was used without further purification. The spectroscopic data of **7** are in agreement with those reported in the literature.^68^

#### *N*,*N*′-((2*S*,3*S*)-1,4-Bis(benzyloxy)butane-2,3-diyl)bis(2-bromoacetamide) (**8**)

Compound **7** (2.6 g, 8.66 mmol) was dissolved in DCM (110 mL) and combined with 50 mL of a 0.4 M aqueous solution of K_2_CO_3_. The mixture was cooled to 0 °C, and bromo acetylbromide (2.5 mL, 27 mmol) was added slowly. For 10 h, the mixture was stirred at 0 °C. The aqueous layer of the reaction mixture was extracted thrice with DCM (3 × 50 mL). The organic layers were all combined and dried with anhydrous Na_2_SO_4_, filtrated, and concentrated under reduced pressure. The crude product was purified by flash silica gel column chromatography (0:100 → 10:90 EtOAc/DCM, *R*_f_ = 0.46 for 20:80 EtOAc/DCM). Product **8** was obtained as a white solid (3.29 g, 6.06 mmol) with a 70% yield. Compound **8**^1^H NMR (400.130 MHz, CDCl_3_, 293 K): δ = 3.46–3.55 (m, 4H, 2CH_2_O), 3.77 (s, 4H, 2CH_2_Br), 4.35–4.39 (m, 2H, 2CHCH_2_O), 4.57 (s, 4H, 2CH_2_Ph), 6.59 (s, 2H, NH), 7.20–7.33 (m, 10H, 2 (CH)_5_C). ^13^C NMR (100.613 MHz, CDCl_3_, 293 K): δ = 51.69 (2CHCH_2_O), 68.65 (2CH_2_O), 73.38 (2CH_2_Ph), 127.93–128.52 (2(CH)_5_C), 137.42 (2C(CH)_5_), 166.06 (2CONH). HR-MS: *m*/*z* 541.032 [M + H]^+^, calcd [C_22_H_26_Br_2_N_2_O_4_] 541.034. FTIR (cm^–1^): 3711.9, 3279.7, 2982.1, 2864.8, 2844.7, 2826.1, 1652.7, 1559.7, 1054.6, 1033.1, 1013.1, 747.0, 698.3. [α]_D_^20^ = −42.27 (*C* = 7.5 mg/mL, CH_2_Cl_2_).

#### (5*S*,6*S*,11*S*,12*S*)-5,6,11,12-Tetrakis((benzyloxy)methyl)-1,4,7,10-tetraazacyclododecane-2,9-dione (**9**)

NaHCO_3_ (1.4 g, 16.67 mmol) was added to 550 mL acetonitrile. Compound **7** (0.5 g, 1.67 mmol) and compound **8** (0.9 g, 1.67 mmol) were added to the mixture, which was then heated to 80 °C, and stirred continuously for 24 h. The reaction mixture was filtrated and concentrated under reduced pressure. The crude product was purified with silica gel column chromatography (0:100 → 10:90 MeOH/DCM, *R*_f_ = 0.49 MeOH/DCM 10:90) to yield 30% of compound **9** (0.26 g, 0.38 mmol) as a white solid. Compound **9**^1^H NMR (400.130 MHz, CD_3_OD, 293 K): δ = 3.34–3.54 (m, 6H, 2CH_2_NH, 2CHCH_2_O), 3.60–3.78 (m, 8H, 4 CH_2_O), 4.33–4.56 (m, 10H, 2CHCH_2_O, 4CH_2_Ph), 4.57 (s, 4H, 2CH_2_O), 6.59 (s, 4H, 4NH), 7.28–7.38 (m, 20H, 4(CH)_5_C). ^13^C NMR (100.613 MHz, CD_3_OD, 293 K): δ = 52.68 (2CH_2_O), 52.95 (2CHCH_2_O), 61.80 (2CHCH_2_O), 67.22 (2CH_2_O), 69.80 (2CH_2_NH), 74.35 (2CH_2_Ph), 74.68 (2CH_2_Ph), 128.99–129.62 (2(CH)_5_C), 138.34 (C(CH)_5_), 138.99 (C(CH)_5_), 168.79 (2CONH). HR-MS: *m*/*z* 681.366 [M + H]^+^, calcd [C_40_H_48_N_4_O_6_] 681.365. FTIR (cm^–1^): 3650.3, 2982.1, 2844.7, 1652.7, 1054.6, 1033.1, 1013.1, 736.9, 696.9, 668.3. [α]_D_^20^ = −24.33° (*C* = 3 mg/mL, MeOH).

#### (2*S*,3*S*,8*S*,9*S*)-2,3,8,9-Tetrakis((benzyloxy)methyl)-1,4,7,10-tetraazacyclododecane (**10**)

Compound **9** (0.208 g, 306 μmol) was co-evaporated three times with dried toluene and dissolved in dried THF (7.7 mL). A solution of LiAlH_4_ (1.0 M in toluene, 10 mL) was cooled down to 0 °C while kept under N_2_ atmosphere. The mixture of compound **9** in THF was dropwise carefully added to the LiAlH_4_ solution, after which the reaction was heated to 80 °C while stirred and kept under N_2_ atmosphere. After 3 h, no starting material **9** was observed by LC–MS. The reaction mixture was cooled to 0 °C and quenched adding 5 mL of H_2_O and 5 mL of 3 M KOH solution. The reaction mixture was filtered over celite and concentrated under reduced pressure. Purification was done over a self-packed C_18_ reverse-phase silica gel column using a gradient of 0–50% ACN in H_2_O (0.2% TFA). Compound **10** was obtained as a white solid (0.183 g, 294 μmol, 95% yield). ^1^H NMR (400.130 MHz, CD_3_OD, 293 K): δ = 2.90 (s, 8H, 4CH_2_NH), 3.23 (b, 4H, 4CHCH_2_O), 3.41–3.67 (q, 8H, 4CH_2_O), 4.40–4.52 (q, 8H, 4CH_2_Ph), 7.30–7.37 (m, 20H, 4(CH)_5_C). ^13^C NMR (100.613 MHz, CD_3_OD, 293 K): δ = 42.16 (4CH_2_NH), 65.68 (4CH_2_O), 74.35 (4CH_2_Ph), 129.19–129.59 (4(CH)_5_C), 138.63 (4C(CH)_5_). HR-MS: *m*/*z* 653.416 [M + H]^+^, calcd [C_40_H_52_N_4_O_4_] 653.416. FTIR (cm^–1^): 3853.5, 3736.2, 3628.9, 2982.1, 2923.4, 2376.8, 1496.8, 1339.4, 1202.0, 1123.3, 1054.6, 1033.1, 1013.1, 741.2. [α]_D_^20^ = −9.09° (*C* = 2.2 mg/mL, CH_2_Cl_2_).

#### Tetra-*tert*-butyl 2,2′,2″,2″′-((2*S*,3*S*,8*S*,9*S*)-2,3,8,9-Tetrakis((benzyloxy)methyl)-1,4,7,10-tetraazacyclododecane-1,4,7,10-tetrayl)tetraacetate (**11**)

Compound **10** (50 mg, 77 μmol) and *tert-*butyl 2-bromoacetate (120 mg, 616 μmol) were dissolved in dried ACN (0.8 mL). K_2_CO_3_ (85 mg, 616 μmol) was added to the solution and stirred at room temperature for 16 h. The solution was filtrated through Celite, concentrated, and purified on silica gel column chromatography (EtOAc/acetone 5:1, *R*_f_ = 0.4) to give compound **11** (60 mg, 54 μmol, 80%) as a white solid. ^1^H NMR (400.130 MHz, CD_3_OD, 293 K): δ = 1.48 (s, 18H, 2(CH_3_)_3_C), 1.49 (s, 18H, 2(CH_3_)_3_C), 2.25–2.28 (d, 2H, ^3^*J* = 12 Hz, CH_2_N), 2.51–2.58 (t, 2H, ^3^*J* = 12 Hz, CH_2_N), 2.63–2.70 (t, 2H, ^3^*J* = 12 Hz, CH_2_N), 2.84–2.88 (q, 2H, 2CHCH_2_O), 3.00–3.05 (d, 2H, ^3^*J* = 12 Hz, CH_2_COOtBu), 3.00–3.10 (2H, CH_2_N), 3.00–3.10 (2H, 2CHCH_2_O), 3.36–3.78 (m, 6H, 2CH_2_COOtBu, CH_2_OBn), 3.52–3.56 (m, 2H, CH_2_OBn), 3.62–3.65 (d, 2H, ^3^*J* = 12 Hz, CH_2_COOtBu), 3.71–3.77 (t, 4H, ^3^*J* = 12 Hz, 2CH_2_OBn), 4.19–4.40 (m, 8H, 4 CH_2_Ph). ^13^C NMR (100.613 MHz, CD_3_OD, 293 K): δ = 28.34 (2C(CH_3_)), 28.40 (2C(CH_3_)), 45.84 (2CH_2_N), 50.52 (2CH_2_N), 54.14 (2CH_2_COOtBu), 54.69 (2CH_2_COOtBu), 56.00 (CHCH_2_O), 60.22 (CHCH_2_O), 65.71 (2CH_2_OBn), 67.15 (2CH_2_OBn), 73.00 (CH_2_Ph), 74.18 (CH_2_Ph), 82.75 (2C(CH_3_)_3_), 82.81 (2C(CH_3_)_3_), 128.90–129.47 (4 (CH)_5_C), 139.11 (2C(CH)_5_), 139.15 (2C(CH)_5_), 174.68 (2COOtBu), 175.45 (2COOtBu). HR–MS: *m*/*z* 1109.68 [M + H]^+^, calcd [C_64_H_92_N_4_O_12_] 1109.68. FTIR (cm^–1^): 3853.5, 3736.2, 3628.9, 2982.1, 2923.4, 2376.8, 1496.8, 1339.4, 1202.0, 1123.3, 1054.6, 1033.1, 1013.1, 741.2. [α]_D_^20^ = −8.1° (*C* = 1 mg/mL, CH_2_Cl_2_).

#### Tetra-*tert*-butyl 2,2′,2″,2″′-((2*S*,3*S*,8*S*,9*S*)-2,3,8,9-Tetrakis(hydroxymethyl)-1,4,7,10-tetraazacyclododecane-1,4,7,10-tetrayl)tetraacetate (**12**)

Compound **11** (0.2 g, 0.18 mmol) was dissolved in 3 mL of ethanol. Pd/C (10% wt Pd on carbon, 20 mg) was added at room temperature, after which the atmosphere was changed to H_2_. The reaction was stirred and monitored using LC–MS analysis. After 72 h, no starting material was detected on LC–MS. The mixture was filtrated through Celite and concentrated. The crude product was purified by gel filtration to afford compound **12** (0.13 mg, 0.14 mmol, 79%) as a white solid. ^1^H NMR (500.130 MHz, D_2_O, 293 K): δ =1.50 (s, 18H, 2 (CH_3_)_3_C), 1.51 (s, 18H, 2 (CH_3_)_3_C) 2.31–2.34 (d, 2H, ^3^*J* = 14.4 Hz, CH_2_N), 2.57–2.62 (t, 2H, ^3^*J* = 10 Hz, CH_2_N), 2.72–2.77 (m, 2H, CH_2_N), 2.72–2.77 (m, 2H, 2CHCH_2_OH), 2.84–2.88 (q, 2H, 2CHCH_2_O), 3.01–3.04 (m, 2H, 2CHCH_2_O), 3.08–3.12 (d, 2H, ^3^*J* = 20 Hz, CH_2_O), 3.08–3.17 (m, 2H, CH_2_N), 3.42–3.56 (q, 4H, CH_2_OH), 3.64–3.68 (d, 2H, ^3^*J* = 20 Hz, CH_2_O), 3.70–3.72 (dd, 2H, ^3^*J* = 3 Hz, CH_2_O), 3.94–3.97 (dd, 2H, ^3^*J* = 3 Hz, CH_2_O), 3.85–3.94 (m, 4H, CH_2_OH). ^13^C NMR (125.758 MHz, CD_3_OD, 293 K): δ = 28.40 (2(CH_3_)_3_C), 28.41 (2(CH_3_)_3_C), 45.39 (2CH_2_N), 50.33 (2CH_2_N), 53.92 (2CH_2_OH), 54.31 (2CH_2_O), 57.62 (2CH_2_O), 57.83 (2CHCH_2_O), 58.97 (2CH_2_OH), 62.20 (2CHCH_2_O), 82.82 (2C(CH_3_)_3_), 82.84 (2C(CH_3_)_3_), 174.66 (2COOtBu), 175.46 (2COOtBu). HR-MS: *m*/*z* 749.492 [M + H]^+^, calcd [C_36_H_68_N_4_O_12_] 749.491. FTIR (cm^–1^): 2983.4, 2923.4, 2866.2, 2380.2, 2310.8, 1555.4, 1506.8, 1456.7, 1054.6, 1033.1, 1013.1. [α]_D_^20^ = −9.8° (*C* = 1 mg/mL, CH_2_Cl_2_).

#### 2,2′,2″,2″′-((2*S*,3*S*,8*S*,9*S*)-2,3,8,9-Tetrakis(hydroxymethyl)-1,4,7,10-tetraazacyclododecane-1,4,7,10-tetrayl)tetraacetic Acid (HMDOTA)

Compound **12** (30 mg, 40 μmol) was dissolved in 1 mol/L HCl (4 mL) and heated at 50 °C under continuous stirring. After 5 h, no starting material or reaction intermediates were observed in LC–MS analysis. The reaction mixture was quenched by adding saturated aqueous NaHCO_3_ to adjust the pH to neutral (circa 3 mL). Purification by gel filtration afforded HMDOTA as a white solid. ^1^H NMR (600.130 MHz, D_2_O, 293 K): δ = 2.69–2.72 (d, 2H, ^3^*J* = 14 Hz, CH_2_NH), 2.94–2.96 (t, 2H, ^3^*J* = 11 Hz, CH_2_N), 3.07–3.08 (b, 2H, 2CHCH_2_OH), 3.22–3.36 (m, 8H, 2CH_2_N, 2CHCH_2_OH, CH_2_COOH), 3.56–3.59 (d, ^3^*J* = 16 Hz, CH_2_COOH), 3.82–4.10 (m, 12H, 4CH_2_OH, 2CH_2_COOH). ^13^C NMR (150.903 MHz, D_2_O, 293 K): δ = 45.47 (2CH_2_N), 51.16 (2CH_2_N), 57.22 (2CH_2_OH), 57.38 (2CH_2_OH), 57.89 (4CH_2_COOH), 58.50 (2CHCH_2_OH), 64.15 (2CHCH_2_OH), 180.50 (COOH), 180.86 (COOH). HR-MS: *m*/*z* 525.241 [M + H]^+^, calcd [C_20_H_36_N_4_O_12_] 525.241. FTIR (cm^–1^): 2983.5, 2923.3, 2866.2, 2380.2, 2310.8, 1555.4, 1506.8, 1456.7, 1054.6, 1033.1, 1013.1. [α]_D_^20^ = −4.3° (*C* = 1 mg/mL, MeOH).

## Supporting Information Available

Intermolecular side reaction; 1D ^1^H spectra of Eu(III)-HMDOTA; 1D ^1^H NMR spectrum of Eu(III)-DOTA complex; 1D ^1^H NMR spectrum of Eu(III)-DO2A-DipyNox complex; globally fitted inversion recovery profile; 1D ^1^H NMR spectrum of Eu(III)-HMDOTA complex; yield of cyclization; and integrals of SAP and TSAP conformers (PDF)

## References

[ref1] Wahsner J.; Gale E. M.; Rodríguez-Rodríguez A.; Caravan P. Chemistry of MRI Contrast Agents: Current Challenges and New Frontiers. Chem. Rev. 2019, 119, 957–1057. 10.1021/acs.chemrev.8b00363. 30350585 PMC6516866

[ref2] Liu W.-M.; Overhand M.; Ubbink M. The Application of Paramagnetic Lanthanoid Ions in NMR Spectroscopy on Proteins. Coord. Chem. Rev. 2014, 273–274, 2–12. 10.1016/j.ccr.2013.10.018.

[ref3] Heffern M. C.; Matosziuk L. M.; Meade T. J. Lanthanide Probes for Bioresponsive Imaging. Chem. Rev. 2014, 114, 4496–4539. 10.1021/cr400477t. 24328202 PMC3999228

[ref4] Cacheris W. P.; Nickle S. K.; Sherry A. D. Thermodynamic Study of Lanthanide Complexes of 1,4,7-Triazacyclononane-N,N′,N″-Triacetic Acid and 1,4,7,10-Tetraazacyclododecane-N,N′,N″,N‴-Tetraacetic Acid. Inorg. Chem. 1987, 26, 958–960. 10.1021/ic00253a038.

[ref5] Baranyai Z.; Brücher E.; Iványi T.; Király R.; Lázár I.; Zékány L. Complexation Properties of N,N′,N″,N‴-[1,4,7,10-Tetraazacyclododecane-1,4,7,10-Tetrayltetrakis(1-Oxoethane-2,1-Diyl)]Tetrakis[Glycine] (H_4dotagl_). Equilibrium, Kinetic, and Relaxation Behavior of the Lanthanide(III) Complexes. HCA 2005, 88, 604–617. 10.1002/hlca.200590042.

[ref6] Spirlet M. R.; Rebizant J.; Desreux J. F.; Loncin M. F. Crystal and Molecular Structure of Sodium Aqua(1,4,7,10-Tetraazacyclododecane-1,4,7,10-Tetraacetato)Europate(III) Tetrahydrate Na^+^(EuDOTA.H_2_O)-.4H_2_O, and Its Relevance to NMR Studies of the Conformational Behavior of the Lanthanide Complexes Formed by The. Inorg. Chem. 1984, 23, 359–363. 10.1021/ic00171a018.

[ref7] Chang C. A.; Francesconi L. C.; Malley M. F.; Kumar K.; Gougoutas J. Z.; Tweedle M. F.; Lee D. W.; Wilson L. J. Synthesis, Characterization, and Crystal Structures of M(DO3A) (M = Iron, Gadolinium) and Na[M(DOTA)] (M = Fe, Yttrium, Gd). Inorg. Chem. 1993, 32, 3501–3508. 10.1021/ic00068a020.

[ref8] Aime S.; Barge A.; Benetollo F.; Bombieri G.; Botta M.; Uggeri F. A Novel Compound in the Lanthanide(III) DOTA Series. X-Ray Crystal and Molecular Structure of the Complex Na[La(DOTA)La(HDOTA)]·10H_2_O. Inorg. Chem. 1997, 36, 4287–4289. 10.1021/ic9704501.

[ref9] Meyer M.; Dahaoui-Gindrey V.; Lecomte C.; Guilard R. Conformations and Coordination Schemes of Carboxylate and Carbamoyl Derivatives of the Tetraazamacrocycles Cyclen and Cyclam, and the Relation to Their Protonation States. Coord. Chem. Rev. 1998, 178–180, 1313–1405. 10.1016/S0010-8545(98)00169-6.

[ref10] Aime S.; Botta M.; Fasano M.; Marques M. P. M.; Geraldes C. F. G. C.; Pubanz D.; Merbach A. E. Conformational and Coordination Equilibria on DOTA Complexes of Lanthanide Metal Ions in Aqueous Solution Studied by ^1^H-NMR Spectroscopy. Inorg. Chem. 1997, 36, 2059–2068. 10.1021/ic961364o. 11669824

[ref11] Aime S.; Botta M.; Ermondi G. NMR Study of Solution Structures and Dynamics of Lanthanide(III) Complexes of Dota. Inorg. Chem. 1992, 31, 4291–4299. 10.1021/ic00047a016.

[ref12] Aime S.; Botta M.; Garda Z.; Kucera B. E.; Tircso G.; Young V. G.; Woods M. Properties, Solution State Behavior, and Crystal Structures of Chelates of DOTMA. Inorg. Chem. 2011, 50, 7955–7965. 10.1021/ic2012827. 21819052 PMC3204394

[ref13] Kumas C.; Fernando W. S.; Zhao P.; Regueiro-Figueroa M.; Kiefer G. E.; Martins A. F.; Platas-Iglesias C.; Sherry A. D. Unexpected Changes in the Population of Coordination Isomers for the Lanthanide Ion Complexes of DOTMA-Tetraglycinate. Inorg. Chem. 2016, 55, 9297–9305. 10.1021/acs.inorgchem.6b01390. 27603690 PMC5221692

[ref14] Jacques V.; Desreux J. F. Quantitative Two-Dimensional EXSY Spectroscopy and Dynamic Behavior of a Paramagnetic Lanthanide Macrocyclic Chelate: YbDOTA(DOTA = 1,4,7,10-Tetraazacyclododecane-N,N′,N″,N‴-Tetraacetic Acid). Inorg. Chem. 1994, 33, 4048–4053. 10.1021/ic00096a033.

[ref15] Desreux J. F. Nuclear Magnetic Resonance Spectroscopy of Lanthanide Complexes with a Tetraacetic Tetraaza Macrocycle. Unusual Conformation Properties. Inorg. Chem. 1980, 19 (5), 1319–1324. 10.1021/ic50207a042.

[ref16] Vlasie M. D.; Comuzzi C.; van den Nieuwendijk A. M. C. H.; Prudêncio M.; Overhand M.; Ubbink M. Long-Range-Distance NMR Effects in a Protein Labeled with a Lanthanide–DOTA Chelate. Chem. Eur. J. 2007, 13, 1715–1723. 10.1002/chem.200600916. 17115462

[ref17] Eichmüller C.; Skrynnikov N. R. Observation of Microsecond Time-Scale Protein Dynamics in the Presence of Ln^3+^ Ions: Application to the N-Terminal Domain of Cardiac Troponin C. J. Biomol. NMR 2007, 37, 79–95. 10.1007/s10858-006-9105-y. 17180551

[ref18] Hass M. A. S.; Liu W.-M.; Agafonov R. V.; Otten R.; Phung L. A.; Schilder J. T.; Kern D.; Ubbink M. A Minor Conformation of a Lanthanide Tag on Adenylate Kinase Characterized by Paramagnetic Relaxation Dispersion NMR Spectroscopy. J. Biomol. NMR 2015, 61, 123–136. 10.1007/s10858-014-9894-3. 25563704

[ref19] Hass M. A. S.; Keizers P. H. J.; Blok A.; Hiruma Y.; Ubbink M. Validation of a Lanthanide Tag for the Analysis of Protein Dynamics by Paramagnetic NMR Spectroscopy. J. Am. Chem. Soc. 2010, 132, 9952–9953. 10.1021/ja909508r. 20586489

[ref20] Stiller J. B.; Otten R.; Häussinger D.; Rieder P. S.; Theobald D. L.; Kern D. Structure Determination of High-Energy States in a Dynamic Protein Ensemble. Nature 2022, 603, 528–535. 10.1038/s41586-022-04468-9. 35236984 PMC9126080

[ref21] Xu D.; Li B.; Gao J.; Liu Z.; Niu X.; Nshogoza G.; Zhang J.; Wu J.; Su X.-C.; He W.; Ma R.; Yang D.; Ruan K. Ligand Proton Pseudocontact Shifts Determined from Paramagnetic Relaxation Dispersion in the Limit of NMR Intermediate Exchange. J. Phys. Chem. Lett. 2018, 9 (12), 3361–3367. 10.1021/acs.jpclett.8b01443. 29864276

[ref22] Rashid H. U.; Martines M. A. U.; Jorge J.; de Moraes P. M.; Umar M. N.; Khan K.; Rehman H. U. Cyclen-Based Gd^3+^ complexes as MRI Contrast Agents: Relaxivity Enhancement and Ligand Design. Bioorg. Med. Chem. 2016, 24, 5663–5684. 10.1016/j.bmc.2016.09.069. 27729196

[ref23] Woods M.; Aime S.; Botta M.; Howard J. A. K.; Moloney J. M.; Navet M.; Parker D.; Port M.; Rousseaux O. Correlation of Water Exchange Rate with Isomeric Composition in Diastereoisomeric Gadolinium Complexes of Tetra(Carboxyethyl)Dota and Related Macrocyclic Ligands. J. Am. Chem. Soc. 2000, 122, 9781–9792. 10.1021/ja994492v.

[ref24] Woods M.; Kovacs Z.; Zhang S.; Sherry A. D. Towards the Rational Design of Magnetic Resonance Imaging Contrast Agents: Isolation of the Two Coordination Isomers of Lanthanide DOTA-Type Complexes. Angew. Chem., Int. Ed. 2003, 42, 5889–5892. 10.1002/anie.200352234. 14673928

[ref25] Tircso G.; Webber B. C.; Kucera B. E.; Young V. G.; Woods M. Analysis of the Conformational Behavior and Stability of the SAP and TSAP Isomers of Lanthanide(III) NB-DOTA-Type Chelates. Inorg. Chem. 2011, 50, 7966–7979. 10.1021/ic2012843. 21819053 PMC3204396

[ref26] Ratnakar S. J.; Woods M.; Lubag A. J. M.; Kovács Z.; Sherry A. D. Modulation of Water Exchange in Europium(III) DOTA–Tetraamide Complexes via Electronic Substituent Effects. J. Am. Chem. Soc. 2008, 130, 6–7. 10.1021/ja076325y. 18067296 PMC2716115

[ref27] Zhang S.; Kovacs Z.; Burgess S.; Aime S.; Terreno E.; Sherry A. D. {DOTA-Bis(Amide)}lanthanide Complexes: NMR Evidence for Differences in Water-Molecule Exchange Rates for Coordination Isomers. Chem. Eur. J. 2001, 7, 288–296. 10.1002/1521-3765(20010105)7:1<288::AID-CHEM288>3.0.CO;2-6. 11205022

[ref28] Bünzli J.-C. G.; Piguet C. Taking Advantage of Luminescent Lanthanide Ions. Chem. Soc. Rev. 2005, 34, 1048–1077. 10.1039/b406082m. 16284671

[ref29] Zapolotsky E. N.; Qu Y.; Babailov S. P. Lanthanide Complexes with Polyaminopolycarboxylates as Prospective NMR/MRI Diagnostic Probes: Peculiarities of Molecular Structure, Dynamics and Paramagnetic Properties. J. Inclusion Phenom. Macrocyclic Chem. 2022, 102, 1–33. 10.1007/s10847-021-01112-3. PMC8582344 34785985

[ref30] Ranganathan R. S.; Pillai R. K.; Raju N.; Fan H.; Nguyen H.; Tweedle M. F.; Desreux J. F.; Jacques V. Polymethylated DOTA Ligands. 1. Synthesis of Rigidified Ligands and Studies on the Effects of Alkyl Substitution on Acid–Base Properties and Conformational Mobility. Inorg. Chem. 2002, 41, 6846–6855. 10.1021/ic025657v. 12470083

[ref31] Ranganathan R. S.; Raju N.; Fan H.; Zhang X.; Tweedle M. F.; Desreux J. F.; Jacques V. Polymethylated DOTA Ligands. 2. Synthesis of Rigidified Lanthanide Chelates and Studies on the Effect of Alkyl Substitution on Conformational Mobility and Relaxivity. Inorg. Chem. 2002, 41, 6856–6866. 10.1021/ic025695e. 12470084

[ref32] Keizers P. H. J.; Desreux J. F.; Overhand M.; Ubbink M. Increased Paramagnetic Effect of a Lanthanide Protein Probe by Two-Point Attachment. J. Am. Chem. Soc. 2007, 129, 9292–9293. 10.1021/ja0725201. 17608481

[ref33] Keizers P. H. J.; Saragliadis A.; Hiruma Y.; Overhand M.; Ubbink M. Design, Synthesis, and Evaluation of a Lanthanide Chelating Protein Probe: CLaNP-5 Yields Predictable Paramagnetic Effects Independent of Environment. J. Am. Chem. Soc. 2008, 130, 14802–14812. 10.1021/ja8054832. 18826316

[ref34] Polášek M.; Rudovský J.; Hermann P.; Lukeš I.; Vander Elst L.; Muller R. N. Lanthanide(III) Complexes of a Pyridine N-Oxide Analogue of DOTA: Exclusive M Isomer Formation Induced by a Six-Membered Chelate Ring. Chem. Commun. 2004, 22, 2602–2603. 10.1039/B409996F. 15543300

[ref35] Stetter H.; Frank W. Complex Formation with Tetraazac Ycloalkane-N, N′, N″, N‴-Tetraacetic Acids as a Function of Ring Size. Angew. Chem., Int. Ed. 1976, 15, 686 10.1002/anie.197606861.

[ref36] Aime S.; Botta M.; Ermondi G.; Fedeli F.; Uggeri F. Synthesis and NMRD Studies of Gadolinium(3+) Complexes of Macrocyclic Polyamino Polycarboxylic Ligands Bearing .Beta.-Benzyloxy-.Alpha.-Propionic Residues. Inorg. Chem. 1992, 31, 1100–1103. 10.1021/ic00032a035.

[ref37] Jacques V.; Gilsoul D.; Comblin V.; Desreux J. F. Rigidified Macrocyclic Lanthanide Chelates for Magnetic Resonance Imaging. J. Alloys Compd. 1997, 249, 173–177. 10.1016/S0925-8388(96)02529-7.

[ref38] Comblin V.; Gilsoul D.; Hermann M.; Humblet V.; Jacques V.; Mesbahi M.; Sauvage C.; Desreux J. F. Designing New MRI Contrast Agents: A Coordination Chemistry Challenge. Coord. Chem. Rev. 1999, 185–186, 451–470. 10.1016/S0010-8545(99)00028-4.

[ref39] Brittain H. G.; Desreux J. F. Luminescence and NMR Studies of the Conformational Isomers of Lanthanide Complexes with an Optically Active Polyaza Polycarboxylic Macrocycle. Inorg. Chem. 1984, 23, 4459–4466. 10.1021/ic00194a012.

[ref40] Dai L.; Jones C. M.; Chan W. T. K.; Pham T. A.; Ling X.; Gale E. M.; Rotile N. J.; Tai W. C.-S.; Anderson C. J.; Caravan P.; Law G.-L. Chiral DOTA Chelators as an Improved Platform for Biomedical Imaging and Therapy Applications. Nat. Commun. 2018, 9, 857 10.1038/s41467-018-03315-8. 29487362 PMC5829242

[ref41] Lee M. D.; Loh C.-T.; Shin J.; Chhabra S.; Dennis M. L.; Otting G.; Swarbrick J. D.; Graham B. Compact, Hydrophilic, Lanthanide-Binding Tags for Paramagnetic NMR Spectroscopy. Chem. Sci. 2015, 6, 2614–2624. 10.1039/C4SC03892D. 29560247 PMC5812434

[ref42] Geraldes C. F. G. C.; Sherry A. D.; Kiefer G. E. The Solution Structure of Ln (DOTP)5– Complexxes. A Comparison of Lanthanide-Induced Paramagnetic Shifts with the MMX Energy-Minimized Structure. J. Magn. Reson. 1992, 97, 290–304. 10.1016/0022-2364(92)90314-W.

[ref43] Vanasschen C.; Bouslimani N.; Thonon D.; Desreux J. F. Gadolinium DOTA Chelates Featuring Alkyne Groups Directly Grafted on the Tetraaza Macrocyclic Ring: Synthesis, Relaxation Properties, “Click” Reaction, and High-Relaxivity Micelles. Inorg. Chem. 2011, 50, 8946–8958. 10.1021/ic2010997. 21859074

[ref44] Anelli P. L.; Beltrami A.; Franzini M.; Paoli P.; Rossi P.; Uggeri F.; Virtuani M. Gd(III) Complexes of Poly(Hydroxymethyl)Substituted Derivatives of 1,4,7,10-Tetraazacyclododecane-1,4,7,10-Tetraacetic Acid. Inorg. Chim. Acta 2001, 317, 218–229. 10.1016/S0020-1693(01)00364-4.

[ref45] Polášek M.; Kotek J.; Hermann P.; Císařová I.; Binnemans K.; Lukeš I. Lanthanide(III) Complexes of Pyridine-N-Oxide Analogues of DOTA in Solution and in the Solid State. A New Kind of Isomerism in Complexes of DOTA-like Ligands. Inorg. Chem. 2009, 48, 466–475. 10.1021/ic801597z. 19086889

[ref46] Tircso G.; Webber B. C.; Kucera B. E.; Young V. G.; Woods M. Analysis of the Conformational Behavior and Stability of the SAP and TSAP Isomers of Lanthanide(III) NB-DOTA-Type Chelates. Inorg. Chem. 2011, 50, 7966–7979. 10.1021/ic2012843. 21819053 PMC3204396

[ref47] Woods M.; Botta M.; Avedano S.; Wang J.; Sherry A. D. Towards the Rational Design of MRI Contrast Agents: A Practical Approach to the Synthesis of Gadolinium Complexes That Exhibit Optimal Water Exchange. Dalton Trans. 2005, 24, 3829–3837. 10.1039/b510778d. PMC2725757 16311635

[ref48] Webber B. C.; Woods M. Structural Analysis of Isomeric Europium(III) Chelates of NB-DOTMA. Inorg. Chem. 2012, 51, 8576–8582. 10.1021/ic3011597. 22809081

[ref49] Opina A. C. L.; Strickland M.; Lee Y.-S.; Tjandra N.; Byrd R. A.; Swenson R. E.; Vasalatiy O. Analysis of the Isomer Ratios of Polymethylated-DOTA Complexes and the Implications on Protein Structural Studies. Dalton Trans. 2016, 45, 4673–4687. 10.1039/C5DT03210E. 26857249 PMC4807635

[ref50] Strickland M.; Schwieters C. D.; Göbl C.; Opina A. C. L.; Strub M.-P.; Swenson R. E.; Vasalatiy O.; Tjandra N. Characterizing the Magnetic Susceptibility Tensor of Lanthanide-Containing Polymethylated-DOTA Complexes. J. Biomol. NMR 2016, 66, 125–139. 10.1007/s10858-016-0061-x. 27659040 PMC6628275

[ref51] Liu W.-M.; Keizers P. H. J.; Hass M. A. S.; Blok A.; Timmer M.; Sarris A. J. C.; Overhand M.; Ubbink M. A pH-Sensitive, Colorful, Lanthanide-Chelating Paramagnetic NMR Probe. J. Am. Chem. Soc. 2012, 134, 17306–17313. 10.1021/ja307824e. 22994925

[ref52] Miao Q.; Liu W.-M.; Kock T.; Blok A.; Timmer M.; Overhand M.; Ubbink M. A Double-Armed, Hydrophilic Transition Metal Complex as a Paramagnetic NMR Probe. Angew. Chem., Int. Ed. 2019, 58, 13093–13100. 10.1002/anie.201906049. PMC6771572 31314159

[ref53] Prudencio M.; Rohovec J.; Peters J. A.; Tocheva E.; Boulanger M. J.; Murphy M. E. P.; Hupkes H.-J.; Kosters W.; Impagliazzo A.; Ubbink M. A Caged Lanthanide Complex as a Paramagnetic Shift Agent for Protein NMR. Chem. Eur. J. 2004, 10 (13), 3252–3260. 10.1002/chem.200306019. 15224334

[ref54] Liu W.-M.; Skinner S. P.; Timmer M.; Blok A.; Hass M. A. S.; Filippov D. V.; Overhand M.; Ubbink M. A Two-Armed Lanthanoid-Chelating Paramagnetic NMR Probe Linked to Proteins via Thioether Linkages. Chem. Eur. J. 2014, 20 (21), 6256–6258. 10.1002/chem.201400257. 24737492

[ref55] Lee M. D.; Dennis M. L.; Swarbrick J. D.; Graham B. Enantiomeric Two-Armed Lanthanide-Binding Tags for Complementary Effects in Paramagnetic NMR Spectroscopy. Chem. Commun. 2016, 52 (51), 7954–7957. 10.1039/C6CC02325H. 27250640

[ref56] Lee M. D.; Dennis M. L.; Graham B.; Swarbrick J. D. Short Two-Armed Lanthanide-Binding Tags for Paramagnetic NMR Spectroscopy Based on Chiral 1,4,7,10-Tetrakis(2-Hydroxypropyl)-1,4,7,10-Tetraazacyclododecane Scaffolds. Chem. Commun. 2017, 53 (99), 13205–13208. 10.1039/C7CC07961C. 29165449

[ref57] Bradshaw J. S.; Krakowiak K. E.; Izatt R. M.; Zamecka-Krakowiak D. J. New High Yield Syntheses of Cyclams Using the Crab-like Cyclization Reaction. Tetrahedron Lett. 1990, 31, 1077–1080. 10.1016/S0040-4039(00)88730-4.

[ref58] Blahut J.; Hermann P.; Tošner Z.; Platas-Iglesias C. A Combined NMR and DFT Study of Conformational Dynamics in Lanthanide Complexes of Macrocyclic DOTA-like Ligands. Phys. Chem. Chem. Phys. 2017, 19, 26662–26671. 10.1039/C7CP05296K. 28960225

[ref59] Ernst R. R.; Bodenhausen G.; Wokaun A.; Redfield A. G. Principles of Nuclear Magnetic Resonance in One and Two Dimensions. Physics Today 1989, 42, 75–76. 10.1063/1.2811094.

[ref60] Perrin C. L.; Dwyer T. J. Application of Two-Dimensional NMR to Kinetics of Chemical Exchange. Chem. Rev. 1990, 90, 935–967. 10.1021/cr00104a002.

[ref61] Hoeft S.; Roth K. Struktur und Dynamik von Lanthanoid-Tetraazacyclododecantetraacetat-(DOTA-)Komplexen in Lösung. Chem. Ber. 1993, 126, 869–873. 10.1002/cber.19931260404.

[ref62] Zapolotsky E. N.; Pershina E. A.; Babailov S. P. NMR Estimation of the Activation Energy of Conformational Dynamics in the [Dy(DOTA)]– Complex: Energetic Manifestation of the Gadolinium Break. Polyhedron 2022, 225, 116071 10.1016/j.poly.2022.116071.

[ref63] Halgren T. A. Merck Molecular Force Field. I. Basis, Form, Scope, Parameterization, and Performance of MMFF94. J. Comput. Chem. 1996, 17, 490–519. 10.1002/(SICI)1096-987X(199604)17:5/6<490::AID-JCC1>3.0.CO;2-P.

[ref64] Chang Y.-K.; Lo H.-J.; Yan T.-H. A Flexible Strategy Based on a C2-Symmetric Pool of Chiral Substrates: Concise Synthesis of (+)-Valienamine, Key Intermediate of (+)- Pancratistatin, and Conduramines A-1 and E. Org. Lett. 2009, 11 (19), 4278–4281. 10.1021/ol9016194. 19711969

[ref65] Kobayashi Y.; Kokubo Y.; Aisaka T.; Saigo K. Hydrogen-Bonding Sheets in Crystals for Chirality Recognition: Synthesis and Application of (2S,3S)-2,3-Dihydroxy- and (2S,3S)-2,3-Dibenzyloxy-1,4-Bis(Hydroxyamino)Butanes. Tetrahedron: Asymmetry 2008, 19 (21), 2536–2541. 10.1016/j.tetasy.2008.11.006.

[ref66] Colera M.; Costero A. M.; Gaviña P.; Gil S. Synthesis of Chiral 18-Crown-6 Ethers Containing Lipophilic Chains and Their Enantiomeric Recognition of Chiral Ammonium Picrates. Tetrahedron: Asymmetry 2005, 16 (15), 2673–2679. 10.1016/j.tetasy.2005.06.039.

[ref67] Hammerschmidt F.; Kaehlig H. Biosynthesis of Natural Products with a Phosphorus-Carbon Bond. 7. Synthesis of [1,1-^2^H_2_]-, [2,2-^2^H_2_]-, (R)- and (S)-[1-^2^H_1_](2-Hydroxyethyl)Phosphonic Acid and (R,S)-[1-^2^H_1_](1,2-Dihydroxyethyl)Phosphonic Acid and Incorporation Studies into Fosfomycin in Streptomyces Fradiae. J. Org. Chem. 1991, 56 (7), 2364–2370. 10.1021/jo00007a022.

[ref68] Scheurer A.; Mosset P.; Saalfrank R. W. Efficient Synthesis of (2R,3R)- and (2S,3S)-2,3-Diaminobutane-1,4-Diol and Their Dibenzyl Ethers. Tetrahedron: Asymmetry 1997, 8 (8), 1243–1251. 10.1016/S0957-4166(97)00086-4.

