# Supplementary material for: Rigidified and Hydrophilic DOTA-like Lanthanoid Ligands: Design, Synthesis, and Dynamic Properties
Source: Inorg Chem. 2023 Feb 17;62(9):3776–87. doi: 10.1021/acs.inorgchem.2c03768 (PMC9996828; doi:10.1021/acs.inorgchem.2c03768)
Supplement: Supplementary file 1 — ic2c03768_si_001.pdf [file ic2c03768_si_001.pdf]

Supporting Information with

Rigidified and hydrophilic DOTA-like lanthanoid ligands: Design, synthesis and dynamic properties

Qing Miao[1,2], René Dekkers[1], Karthick Babu Sai Sankar Gupta[1], Mark Overhand[1], Rubin Dasgupta[1,3,+], Marcellus Ubbink[1,+]

[1] Leiden Institute of Chemistry, Gorlaeus Laboratories, Leiden University, Einsteinweg 55, 2333 CC Leiden, The Netherlands

[2] College of Chemistry and Chemical Engineering, Key Laboratory of Chemical Additives for China National Light Industry, Shaanxi University of Science and Technology, Xi'an 710021, China

[3] Karolinska Institutet, Dept. of Medical Biochemistry & Biophysics, Solnavägen 9, 17177 Stockholm, Sweden

[+]Correspondence authors: Marcellus Ubbink (m.ubbink@chem.leidenuniv.nl, ORCID 0000-0002-2615-6914) and Rubin Dasgupta (rubin.dasgupta@ki.se, ORCID 0000-0003-4505-7775)

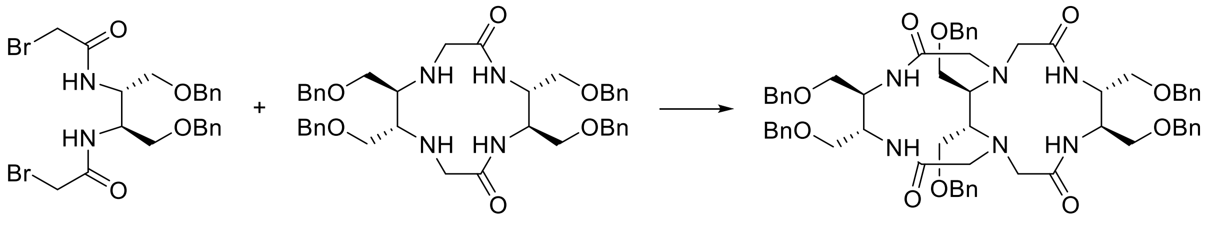

Scheme S1. The intermolecular side reaction discussed in the text.

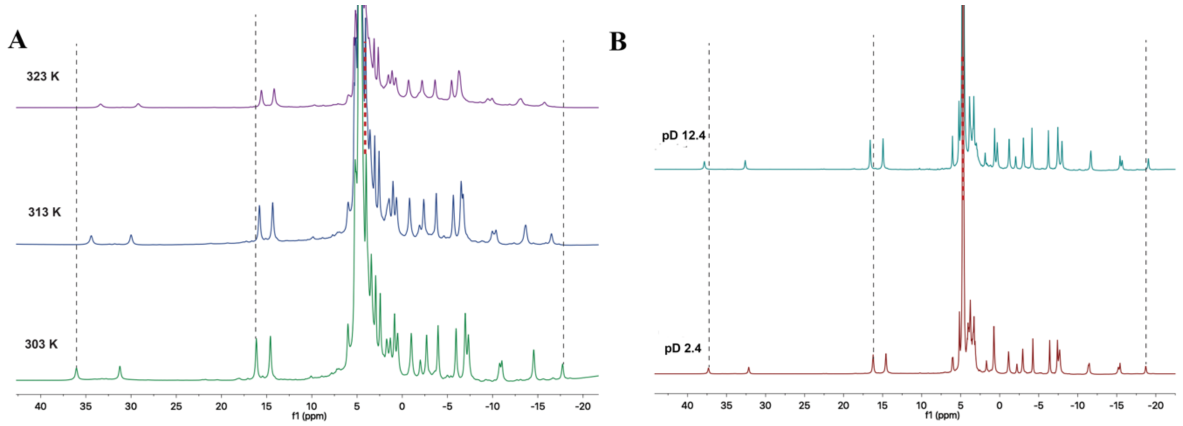

Figure S1. 1D ^{1}H spectra of Eu(III)-HMDOTA. (A, B) at different temperatures (A) and pD (B). Red dashed lines in A and B indicate the reference peaks of methanol. Black dashed lines in A and B act as references to clarify the shifts of the peaks.

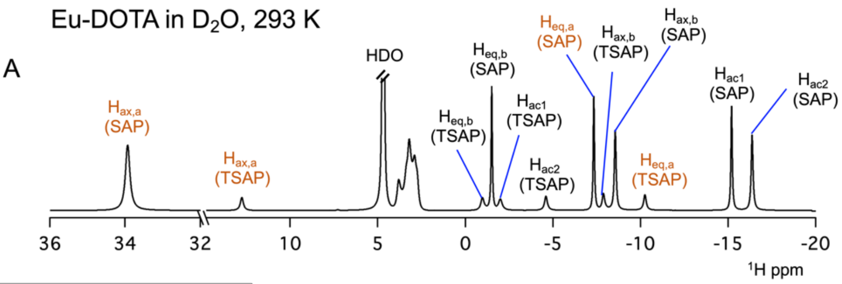

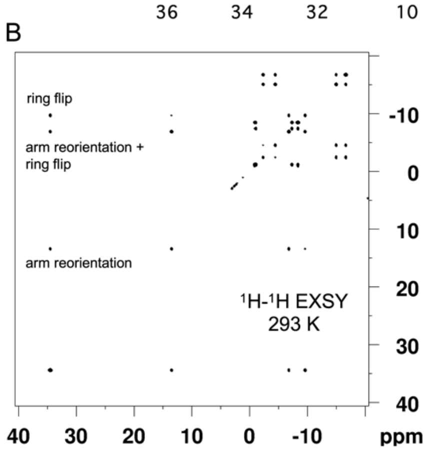

C

	Chemical Shift (ppm)	
	SAP (1, 4)	TSAP (2, 3)
$H_{ax,a}$	33.94	12.76
$H_{eq,a}$	-7.33	-10.24
$H_{ax,b}$	-8.55	-7.88
$H_{eq,b}$	-1.50	-0.97
H_{ac1}	-15.20	-2.20
H_{ac2}	-16.36	-4.60

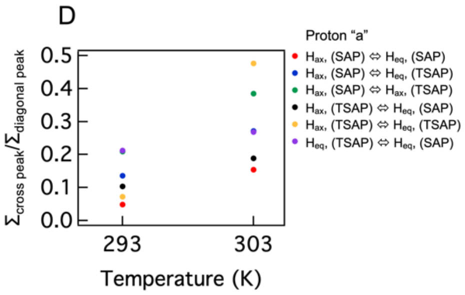

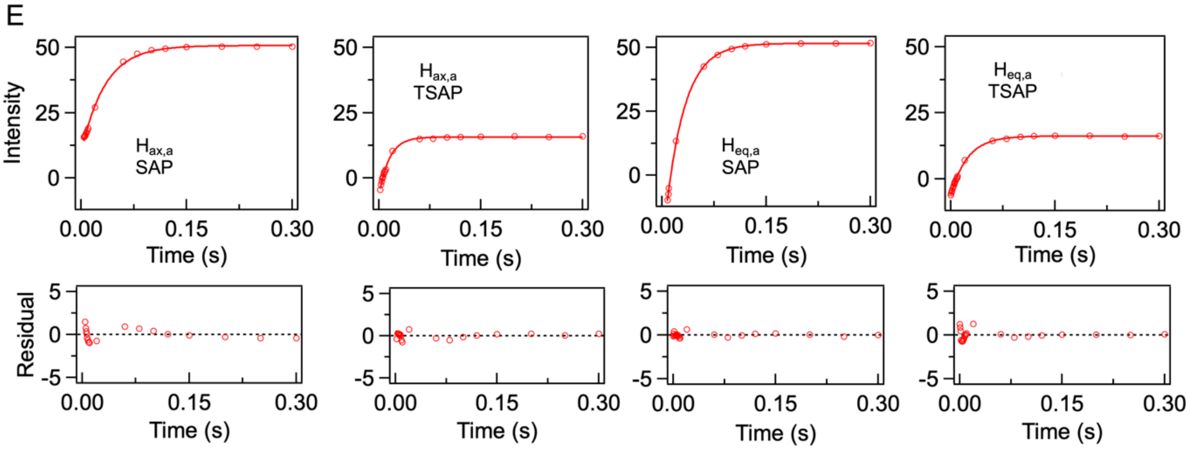

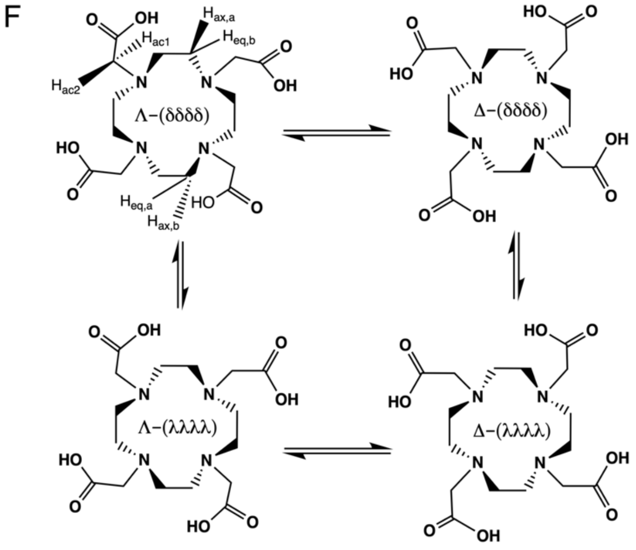

Figure S2. A) 1D ^{1}H NMR spectrum of Eu(III)-DOTA complex in D_2O at 293 K. The assignments of the resonances are shown. The assignments in brown were used for the fitting of 4-state model (see main text for more details). The spectrum is clipped between 32 and 12 ppm because there are no signals in this region. B) ^{1}H-^{1}H EXSY spectrum of Eu(III)-DOTA at 293 K. The cross-peak from $H_{ax,a}$ (SAP1) is assigned to show the exchanging states shown in Figure 1 in the main text. C) Chemical shift in ppm of the resonances in panel A. D) Temperature dependence of the cross-peak integral normalized with the diagonal-peak integral of the four exchanging states. All the exchanging pairs are color coded. E) The inversion recovery curve of the signals marked brown in panel A. The data points are shown as red circles and the solid line is the global fit. The residuals are shown below the graphs. The R_1 relaxation rate estimated was 38 ± 1 s^{-1}. F) Different conformers in DOTA with the studied protons denoted.

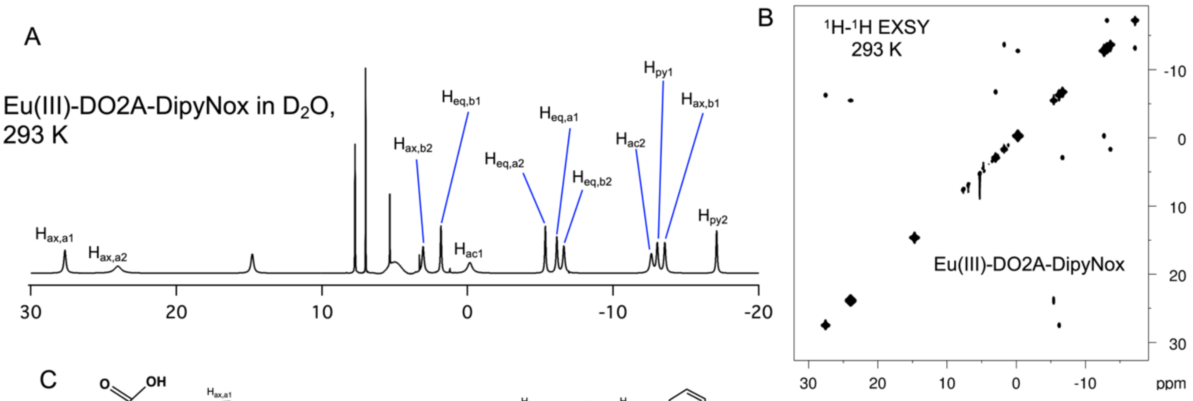

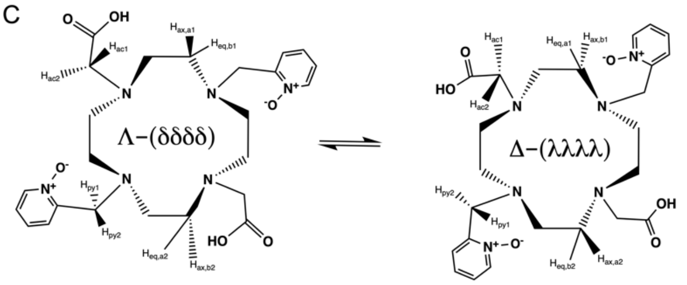

D

	Chemical Shift (ppm) 293 K	
$H_{ax,a1}$ <> $H_{eq,a1}$	27.57	-6.18
$H_{ax,a2}$ <> $H_{eq,a2}$	23.94	-5.42
$H_{ax,b2}$ <> $H_{eq,b2}$	2.97	-6.69
$H_{eq,b1}$ <> $H_{ax,b1}$	1.75	-13.62
H_{ac1} <> H_{ac2}	-0.23	-12.69
H_{py1} <> H_{py2}	-13.12	-17.16

Figure S3. A) 1D ^1H NMR spectrum of Eu(III)-DO2A-DipyNox complex in D_2O at 293 K. The assignments of the resonances are shown. B) ^1H-^1H EXSY spectrum at 293 K. C) Schematic representation of the exchange process in Eu(III)-DO2A-DipyNox complex. D) Chemical shift in ppm of the resonances assigned in panel A.

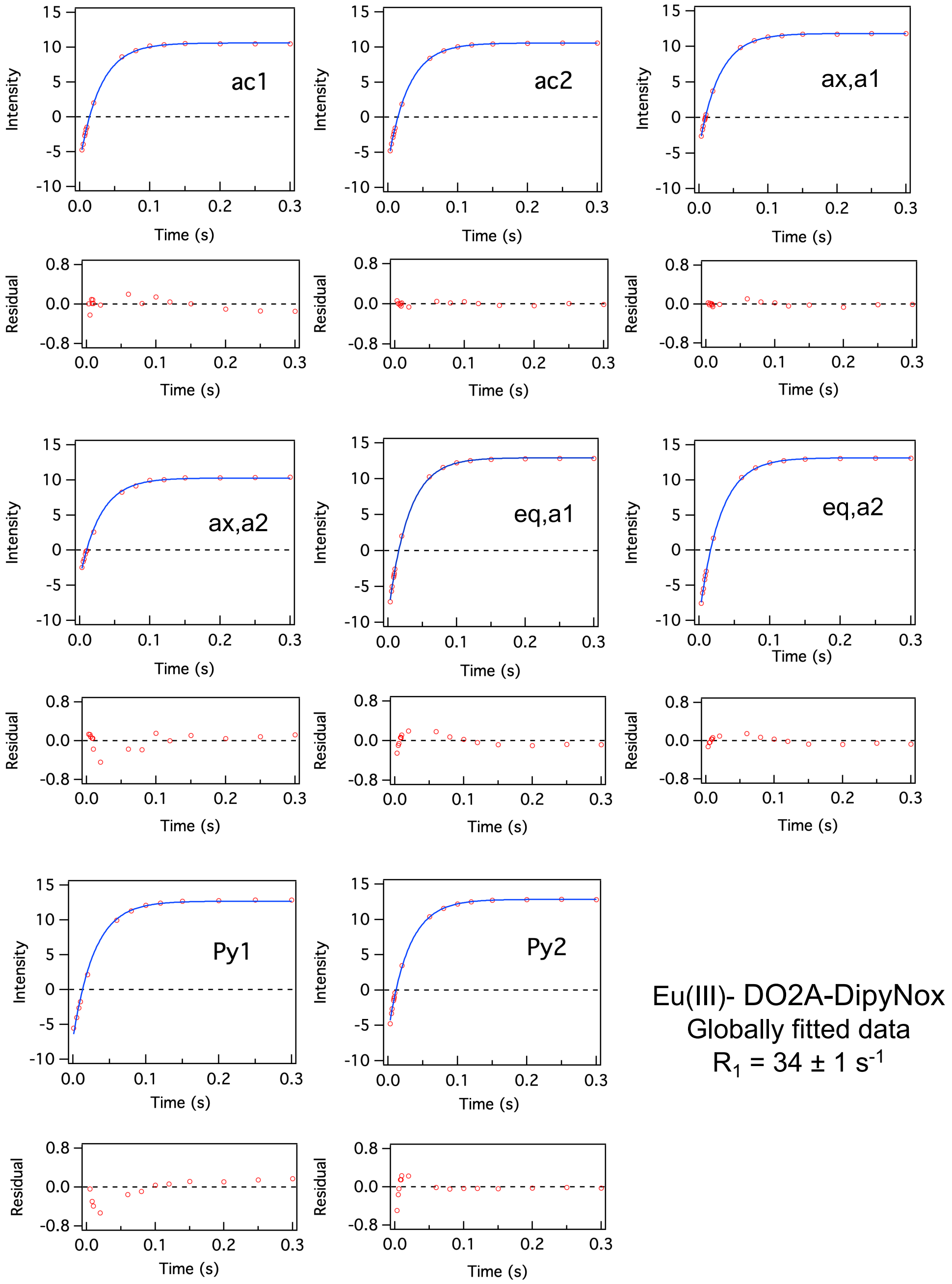

Figure S4. Globally fitted inversion recovery profile of selected ^{1}H resonances from Eu(III)-DO2A-DipyNox with the residuals below each profile. The ^{1}H annotations match those in Figure S3A.

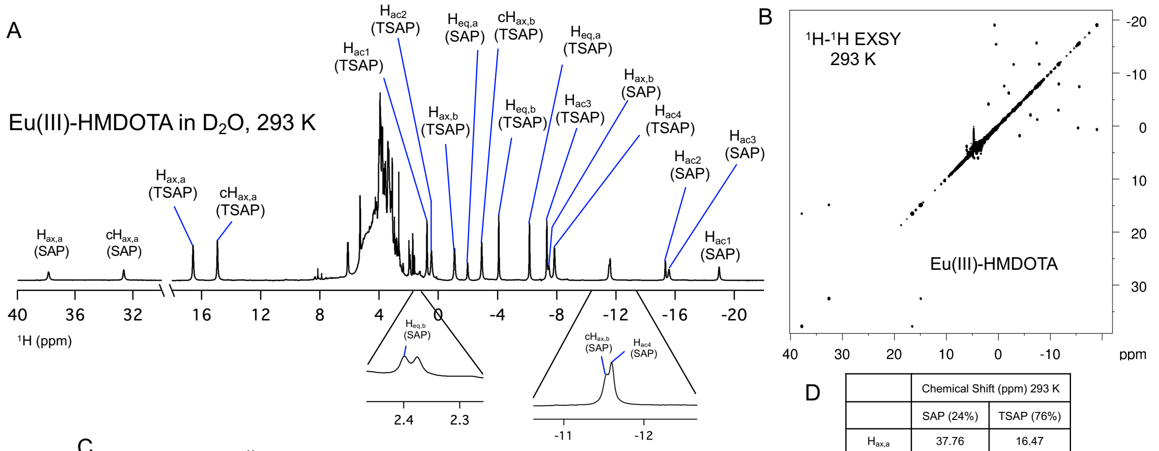

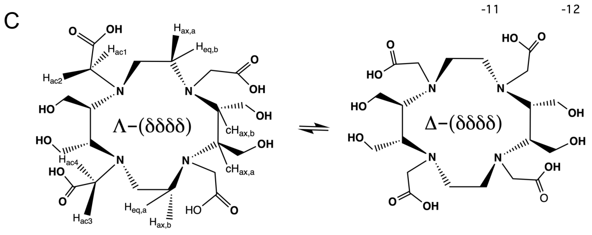

D

	Chemical Shift (ppm) 293 K	
	SAP (24%)	TSAP (76%)
$H_{ax,a}$	37.76	16.47
$H_{eq,a}$	-2.05	-6.24
$H_{ax,b}$	-7.55	-1.17
$H_{eq,b}$	1.84	-4.16
H_{ac1}	-19.05	0.69
H_{ac2}	-15.42	0.39
$cH_{ax,a}$	32.56	14.87
$cH_{ax,b}$	-11.62	-3.00
H_{ac3}	-15.66	-7.40
H_{ac4}	-11.68	-7.92

Figure S5. A) 1D ^{1}H NMR spectrum of Eu(III)-HMDOTA complex in D_2O at 293 K. B) ^{1}H-^{1}H EXSY spectrum of the Eu(III)-HMDOTA complex at 293 K. C) Schematic representation of the exchange process in the Eu(III)-HMDOTA complex. D) Chemical shift in ppm of the resonances assigned in panel A.

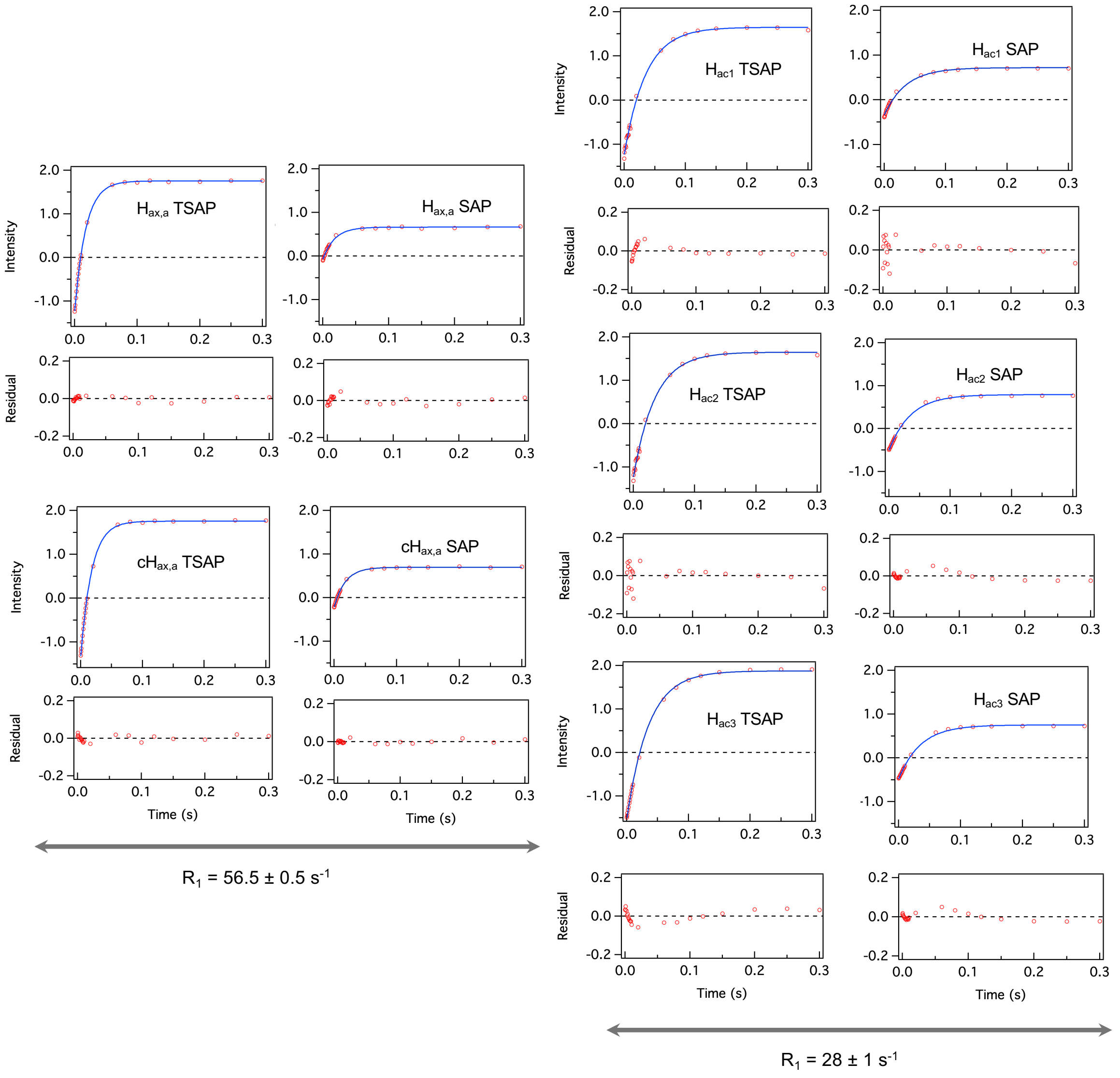

Figure S6. Globally fitted inversion recovery profile of ^{1}H resonances from Eu(III)-HMDOTA with the residuals below each profile. The data are shown as red circle with blue fit line. The panels are arranged as the exchanging pairs from left to right. The exchanging pairs of H_{ax}-TSAP/SAP and cH_{ax}-TSAP/SAP were fitted globally to give a R_1 rate of 56.5 ± 0.5 s^{-1}, while the exchanging pairs for protons H_{ac1}, H_{ac2}, and H_{ac3} were globally fitted separately to give a R_1 rate of 28 ± 1 s^{-1}. The ^{1}H annotations match those in Figure S5A.

Table S1. Yield of cyclization under different conditions.

Temperature (time)	K_2CO_3		$CeCO_3$		Na_2CO_3		$NaHCO_3$	
	a	b	a	b	a	b	a	b
RT (72 hrs)	15%	30%	14%	30%	16%	30%	10%	8%
50 °C (24 hrs)	13%	32%	12%	28%	15%	30%	16%	14%
80 °C (24 hrs)	14%	40%	18%	35%	23%	35%	30%	20%

a: Desired cyclized product, b: Detected side product.

Table S2. Integrals of SAP and TSAP conformers in different compound studies complexed with either Eu(III) or Yb(III). In Yb(III) complexes the coordinated water is expected to be absent.

Compound	Eu (III)		Yb (III)	
	SAP	TSAP	SA’	TSA’
DOTA	1	0.22	1	0.18
DO2A-DipyNox	1	0	1	0
HMDOTA	0.13	1	0.06	1
